# Genome-wide DNA copy number analysis in clonally expanded human ovarian cancer cells with distinct invasive/migratory capacities

**DOI:** 10.18632/oncotarget.14767

**Published:** 2017-01-20

**Authors:** Lei Li, Huimin Bai, Jiaxin Yang, Dongyan Cao, Keng Shen

**Affiliations:** ^1^ Department of Obstetrics and Gynecology, Peking Union Medical College Hospital, Chinese Academy of Medical Sciences & Peking Union Medical College, Beijing, China

**Keywords:** ovarian cancer, tumor heterogeneity, invasion/migration, copy number variation (CNV), genetic targets

## Abstract

Ovarian cancer has the worst prognosis of any gynecological malignancy, and generally presents with metastasis at advanced stages. Copy number variation (CNV) frequently contributes to the alteration of oncogenic drivers. In this study, we sought to identify genetic targets in heterogeneous clones from human ovarian cancers cells. We used array-based technology to systematically assess all the genes with CNVs in cell models clonally expanded from A2780 and SKOV3 ovarian cancer cell lines with distinct highly and minimally invasive/migratory capacities. We found that copy number alterations differed between matched highly and minimally invasive/migratory subclones, differentially affecting specific functional processes including immune response processes, DNA damage repair, cell cycle and cell proliferation. We also identified seven genes as strong candidates, including *DDB1*, *ERCC1*, *ERCC2*, *PRPF19*, *BCAT1*, *CDKN1B* and *MARK4*, by integrating the above data with gene expression and clinical outcome data. Thus, by determining the molecular signatures of heterogeneous invasive/migratory ovarian cancer cells, we identified genes that could be specifically targeted for the treatment and prognosis of advanced ovarian cancers.

## INTRODUCTION

Ovarian cancer is the most lethal gynecologic malignancy, accounting for more than 150,000 deaths annually worldwide [1]. The reason for the poor prognosis of ovarian cancer is that the majority of patients present an advanced stage of this disease, characterized by metastasis to the peritoneal cavity. Importantly, intra-tumor heterogeneity has been reported for these types of tumors [2, 3]. Tumors are thought to originate from a single clonal state that then expands clonally, accompanied by genetic changes that give rise to functional differences, resulting in different stages and characteristics of neoplastic development [4, 5].

Copy number variation (CNV) is increasingly linked to the genetic and phenotypic diversity among cancers, and is frequently associated with the activation of oncogenic drivers or the deletion of tumor suppressors [6–9]. Using either conventional metaphase chromosome-based comparative genomic hybridization [10, 11] or array-based high-resolution genomic technology for identifying genome-wide CNVs in ovarian cancer [12–19], previous studies have identified regions of frequently increased copy number along 1q, 3q26, 7q32–q36, 8q24, 17q32 and 20q13, and reduced copy number along 1p36, 4q, 13q, 16q, 18q and Xq12. In addition, a number of high-level amplifications have been highlighted as predictive biomarkers, including those of *CCNE1*, *RB1*, *MYC*, *ERBB2*, *PIK3CA*, *EVI1*, *AKT2*, *NOTCH3* and *FGFR1*. While studies of numerous patient cohorts have enabled the precise characterization of the genetic alterations that predict clinical outcomes [19, 20] or chemoresistance [12], as well as the precise comparison of the genetic alterations between primary and metastatic lesions [15] or histotype-specific ovarian cancers [21], there has been little effort to correlate clinical outcomes with the genetic alterations of ovarian cancer cell subclones characterized by distinct invasive/migratory capacities. Considering the high malignant potential and poor 5-year survival rate associated with this type of cancer, the mechanisms underlying advanced ovarian cancer should be elucidated through comparison of the genetic profiles of heterogeneous neoplastic subclones.

We previously established stable cellular subclones derived from the human epithelial ovarian cancer cell lines A2780 and SKOV3, which exhibit distinct invasive/migratory capacities [22]. A-H and S-H cells (A2780 and SKOV3 subclones with higher invasive/migratory capacities) exhibited enhanced proliferative, anti-apoptotic and anti-anoikic activity and reduced autophagic activity compared with A-L and S-L cells (A2780 and SKOV3 subclones with lower invasive/migratory capacities). In addition, A-H and S-H cells were significantly more resistant to cisplatin and Taxol *in vitro* and had higher capacities for tumor formation *in vivo* than A-L and S-L cells [22]. These two pairs of subclones with the same hereditary background served as models of intra-tumor heterogeneity. In the present study, we searched for differences in the genomic CNVs of the chosen subclones. Determining the genetic and molecular events leading to the distinct invasive/migratory capacities of these subclones will improve the accuracy of clinical interpretations and the effectiveness of therapeutics for advanced ovarian cancer.

## RESULTS

### Validation of the CNV data

We identified two pairs of subclones derived from the ovarian cancer cell lines A2780 and SKOV3 in our previous work [22]. A-H and S-H cells had higher invasive/migratory capacities than A-L and S-L cells, respectively. We also found that A-H and S-H cells showed enhanced proliferative and anti-apoptotic activities compared with A-L and S-L cells. Moreover, they had higher level of resistance to cisplatin and Taxol *in vitro* and tumor formation capacity *in vivo* [22]. Affymetrix CytoScan™ HD microarrays were used to investigate regions of DNA with copy number alterations for the four subclones. For validation of the array data, we selected several regions for quantitative PCR analysis of A-H versus A-L copy number and S-H versus S-L copy number.

In the A-H versus A-L validation, the relative gene copy numbers in regions of 11q12.2, 12p13.1, 12p12.1 and 19q13.32 of A-H were found to be amplified, whereas the relative gene copy numbers in regions of 4q25, 5q21.3, 5q22.2, 5q31.2, 5q33.3, 9q34.12, 9q34.3 and 9q22.33 of A-H revealed deletion, when the copy number of A-L was set as 1. In contrast, when the gene copy number of A-H was set as 1, the copy numbers in regions of 2q32.3, 2q32.2 and 15q25.1 of A-L were amplified. For S-H/S-L validation, regions of 11q12.1, 12p13.1, 12p12.1 and 19q13.32 of S-H were amplified and regions of 8p23.3 and 17p13.1 of S-H were deleted relative to S-L. In contrast, in S-L cells, regions of 2p14, 3p21.31, 10q24.32, 10q26.3, 15q11.2, 15q15.2 and 15q22.31 were amplified and regions of 8p12 and 8p11.23 were deleted relative to S-H (Supplementary Figure 1). The relative copy numbers agreed with the array data.

### Copy number profiling of the heterogeneous invasive/migratory subclones

**Figure 1:**
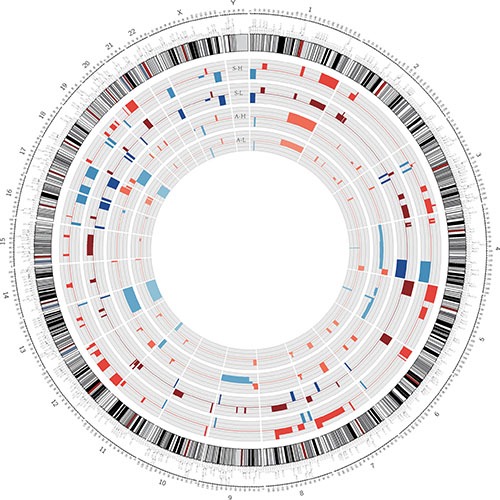
Genetic heterogeneity of the distinct highly and minimally invasive/migratory subclones Circos plot of segmented CNVs in S-H/S-L and A-H/A-L cells. Colored bands expanding toward the center or the periphery of the diagram represent copy number losses or gains, respectively (red, gain; blue, loss).

We compared the genomic DNA copy numbers of highly and minimally invasive/migratory subclones with a HapMap control set, to determine specific amplifications and deletions in cancer cell lines versus normal samples. The CNV profiles for the subclones are shown in Figure 1. The distributions of altered regions were quite different in the A2780- and SKOV3-derived subclones. In each cell line, a large number of chromosomal differences revealed some degree of genetic heterogeneity between A-H and A-L, S-H and S-L. Encouragingly, the majority of regions agreed with those previously published in studies of ovarian cancer [15, 17–19]. These included amplifications in 1q, 7q35-36, 17q and 20q and deletions in 4q, 5q, 13q, 16q and 18q, among others, in both A-H and A-L cells, as well as amplifications in 1q, 3q, 6p, 7q35-36, 8q, 12p and 20q and deletions in 1p36, 4q, 16q, 17p, 17q, 22q and Xq, among others, in both S-H and S-L cells. It was clear from our analysis that there were fewer copy number changes in the A2780-derived subclones than in the SKOV3-derived subclones. According to previous studies on histotype-specific CNVs in ovarian cancer [21, 23], ovarian serous cancer is characterized by 1q, 3q, 6p, 7q, 8q, 11q, 12p and 20q amplification and 1p36, 4q, 5q, 6q, 8p, 11p, 13q, 15q, 16q, 17, 18q, 22q and X deletion relative to other subtypes. Apparently, the SKOV3-derived subclones were more molecularly similar to ovarian serous cancer than were the A2780-derived subclones.

In the comparison of A-H and A-L, while the CNVs of both A-H and A-L overlapped significantly with those identified in previous studies, large regions were different between the two subclones. Segments of gains in 9p22.3-9p22.2, 11q12.1-11q12.2, 12p13.31-12p11.23, 17q25.2, 18p11.21, 18q11.1-18q11.2, 19q13.32 and 20q13.33 appeared only in A-H (named “A-H-specific gain”), whereas segments of gains in 2p14, 2q32.1, 2q32.2-2q32.3, 15q25.1-15q25.2 and 15q25.3 were observed only in A-L (named “A-L-specific gain”). While we also observed losses in segments of A-H only (named “A-H-specific loss”), we did not find any chromosomal losses in A-L only. Segment locations and genes with CNVs, both shared and specific, are documented in Supplementary Table 4.

Functional analysis by DAVID [24, 25] indicated that the proteins encoded by the “A-H-specific gain” genes (245 genes) were involved in sensory perception, signal transduction, defense responses, nucleotide excision repair, DNA damage removal and cell proliferation. The proteins encoded by the “A-H-specific loss” genes (2313 genes) displayed significant enrichment for cell adhesion and extracellular structure organization. The loss of such genes may promote the epithelial-mesenchymal transition, whereby cancer cells lose their polarity and adhesive abilities and acquire migratory and invasive capacities that facilitate their detachment from the primary tumor and invasion through the basement membrane into the circulation to remote sites [26–28]. Thus, the loss of these genes could explain the higher invasive/migratory capacities of A-H cells. The “A-L-specific gain” genes (55 genes) pertained mainly to cell apoptosis and death. Copy number gains in these genes might contribute to the lower capacity for proliferation and invasion/migration and the higher rate of apoptosis of the A-L subclone (Supplementary Table 5).

In the S-H and S-L comparison, segments of gains that appeared only in S-H were distributed across 3p, 5q, 6p, 7q, 8p, 11, 12, 14q, 16p, 17, 19q and 20q (named “S-H-specific gain”), while segments of losses only in S-H were located on 8p23.3-8p23.2, 8p21.3-8p21.2, 8q24.12, 17p13.1-17p11.2 and Xq21.31 (named “S-H-specific loss”). S-L also harbored specific gains in 2p14, 2p16.1, 3p21.31, 3q25.31, 6p25.3, 8q24.12, 9p11.2, 9q21.11, 9q13, 10q24.32-10q26.3, 11q24.2, 15q11.2-15q25.2 and 16p11.2 and specific losses in 7q31.1, 8p12-8p11.23 and 8p11.23 (named “S-L-specific gain” and “S-L-specific loss,” respectively; Supplementary Table 6).

GO analysis of the genes within regions specific to S-H and S-L was also performed. The GO terms and KEGG enrichment results are listed in Supplementary Table 7. The genes with “S-H-specific gain” (1520 genes) included the Wnt/β-catenin signaling pathway members *WNT2* and *CAV1*; *CAV2*, the product of which is likely to be involved in signal transduction, cellular growth and apoptosis; the cell-cycle-related gene *CDKN1B*; and cortactin binding protein 2 (*CTTNBP2*). The proteins encoded by these genes could function in microtubule polymerization, protein complex assembly and vasodilation. Other notable GO terms with *P* values < 0.05 include the following: cell-cell signaling, regulation of growth, nucleotide excision repair, DNA damage removal, negative regulation of apoptosis, negative regulation of programmed cell death, and negative regulation of cell adhesion. The identification of such pathways seems consistent with the high level of proliferation and low level of apoptosis in S-H cells. Moreover, KEGG analysis revealed significant enrichment of genes with products involved in Melanoma and Glioma (*P* = 0.013 and 0.039) and a trend for those involved in Prostate cancer and Non-small lung cancer (*P* = 0.059 and 0.098). In contrast, the proteins encoded by the “S-H-specific loss” genes (182 genes) appeared to be involved in apoptosis (hsa04210, *P* = 0.002), consistent with the low level of apoptosis among S-H cells. Major functional categories for “S-L-specific gains” (836 genes) included cell death and apoptosis, regulation of the cell cycle, and regulation of cell-matrix adhesion. “S-L-specific losses” (22 genes) were enriched for the regulation of protein binding. The annotations for specific gains/losses in S-H/S-L differed from those of A-H/A-L, demonstrating the heterogeneity of ovarian cancer cell lines. This also suggested that genetic alterations might contribute to functional differences in the two pairs of heterogeneous subclones.

By using matched highly and minimally invasive/migratory subclones, we could also detect regions that were altered in both subclones and further changed in one subclone. These included, for instance, amplifications that were shared between both high and low subclones relative to a normal baseline, and were further amplified in the high or low subclone compared to the other (Supplementary Table 8). For consistency, we focused our analysis on the above results. In the case of shared amplifications in A-H and A-L that were further amplified in A-H cells, we also observed enrichment of genes encoding proteins that were involved in sensory perception, the immune response and signal transduction. Genes amplified in both S-H and S-L and further amplified in S-H were involved in the immune response and growth, and KEGG analysis also revealed a trend in pathways in cancer. We also observed that *ECM1* and *AKT3*, members of the PI3K/AKT/mTOR pathway, as well as *RASSF5*, *RYR2* and *IGFBP3*, were amplified in both high and low subclones relative to normal cells, and also amplified in both A-H and S-H compared with A-L and S-L, consistent with our previous gene expression data [22].

### Analysis of genes identified in CNV regions in both highly or both minimally invasive/migratory subclones

**Figure 2:**
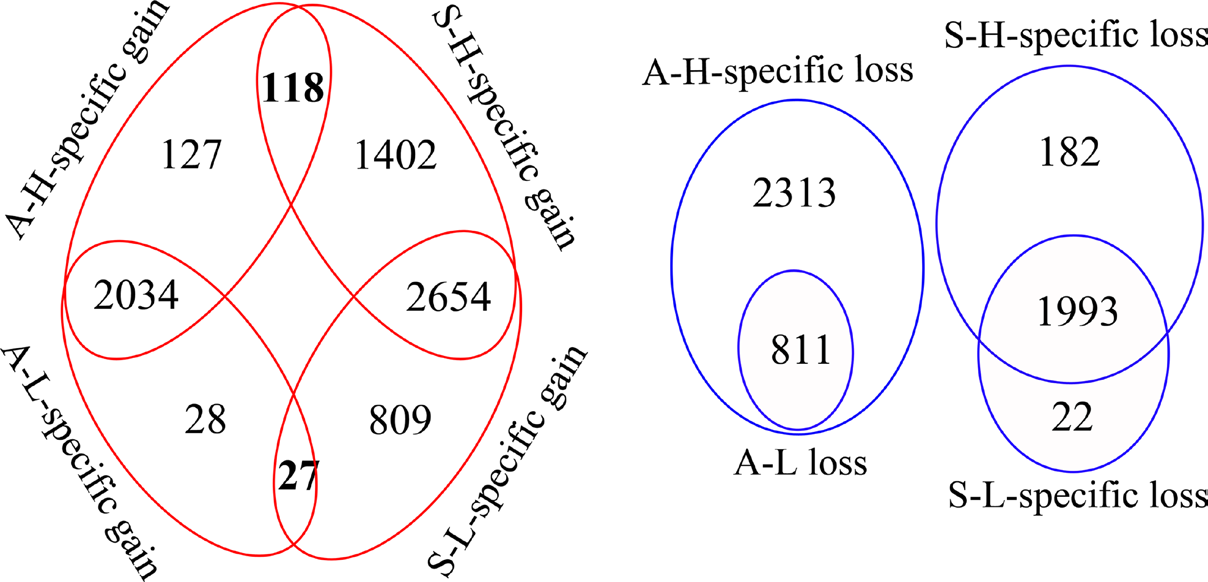
Diagrams indicating the overlapping genes with CNV between the distinct invasive/migratory subclones The red circle indicates the number of amplified genes and the blue circle indicates the number of deleted genes. The red circle demonstrates that there were 245 (117+118) genes amplified as “A-H-specific gains” and 1520 (1402+118) genes amplified as “S-H-specific gains,” with 118 genes overlapping between them. There were 55 (28+27) genes amplified as “A-L-specific gains” and 836 (809+27) genes amplified as “S-L-specific gains,” with 27 genes overlapping between them. The blue circle demonstrates that there were 2313 genes deleted as “A-H-specific losses” and 182 genes deleted as “S-H-specific losses,” as well as 22 genes deleted as “S-L-specific losses”.

Although both pairs of subclones had cell-line-specific characteristics, comparison data between A-H and A-L overlapped to some extent with comparison data between S-H and S-L. There were 118 genes amplified in both “A-H-specific gains” and “S-H-specific gains,” and 27 genes amplified in both “A-L-specific gains” and “S-L-specific gains.” However, there was no gene deleted in both “A-H-specific losses” and “S-H-specific losses,” or in both “A-L-specific losses” and “S-L-specific losses” (Figure 2).

**Figure 3:**
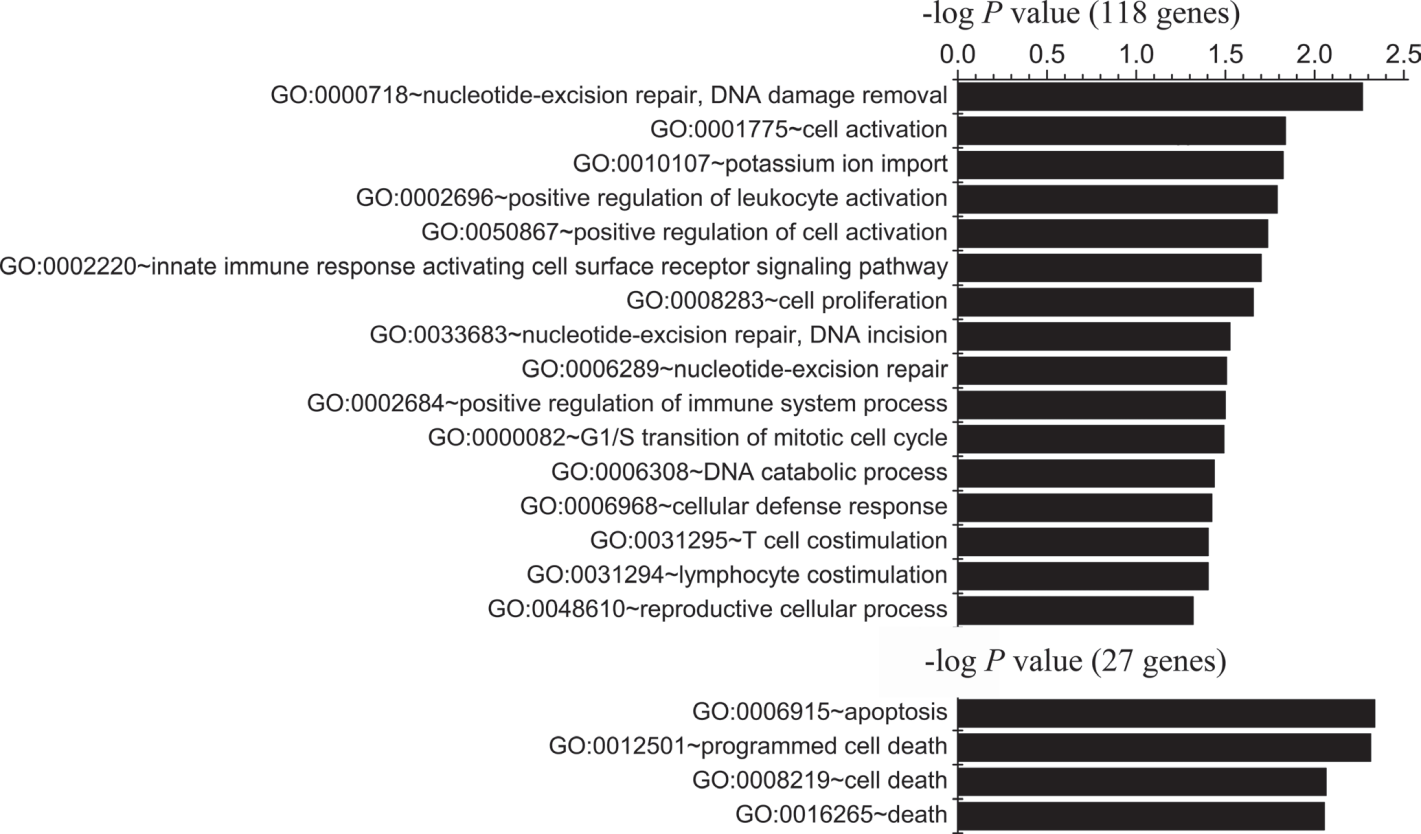
Functional enrichment analysis of genes identified in CNV regions in both highly or both minimally invasive/migratory subclones Gene Ontology-based annotation was used for functional enrichment analysis of genes with shared A-H/S-H-specific gains (118 genes) and genes with shared A-L/S-L-specific gains (27 genes) through DAVID. The bar represents the -log of the *P* value for the significance of enrichment. Only annotations with significant *P* values < 0.05 are shown.

Functional analysis of genes within regions with specific gains for both A-H and S-H (118 genes) revealed enrichment mainly in genes with products involved in defense response (10 genes, *P* = 0.0035), nucleotide excision repair, DNA damage removal (3 genes, *P* = 0.0054), cell activation (6 genes, *P* = 0.0146), regulation of immune system processes (positive regulation of leukocyte activation, 4 genes, *P* = 0.0162; innate immune response activating cell surface receptor signaling pathway, 2 genes, *P* = 0.02; positive regulation of immune system process, 5 genes, *P* = 0.0317; T cell and lymphocyte costimulation, 2 genes, *P* = 0.0395), cell proliferation (7 genes, *P* = 0.0221) and G1/S transition of mitotic cell cycle (3 genes, *P* = 0.0322). Shared A-L- and S-L-specific gains (27 genes) were enriched for genes encoding proteins involved in cell apoptosis and death. The top annotations with *P* values < 0.05 were the following: apoptosis (5 genes, *P* = 0.0046), programmed cell death (5 genes, *P* = 0.0049), cell death (5 genes, *P* = 0.0082), and death (5 genes, *P* = 0.0088) (Figure 3, Supplementary Table 9).

### Assessment of candidate gene expression and patient outcomes

Because the primary purpose of this study was to identify genes that were distinctively altered in highly and minimally invasive/migratory subclones and had a functional effect on tumor metastasis, all genes that were exclusively amplified in both A-H and S-H (relative to A-L and S-L) were analyzed. In the gene ontology analysis, there were both A-H- and S-H-specific gains in a signature of immune genes, including defense response genes, immune system regulatory genes, and immune cell activation genes. These results were consistent with the results of a previous study of serous and endometrioid tumors mainly from the Australian Ovarian Cancer Study [29]. These investigators defined a subtype of high-grade ovarian cancer characterized by up-regulation of immune response genes, with worse survival (both overall survival (OS) and progression-free survival (PFS), *P* < 0.001) than endometrioid ovarian tumors or serous tumors of low grade, early stage and low malignant potential. Another previous study identified amplified regions mapping to 12p12.1, 19q12, 20q11.21 and 20q13.12 with significantly worse outcomes (OS, *P* = 0.0028; PFS, *P* < 0.001) [20]. For comparison to our data, the region of 12p12.1 amplified in both A-H- and S-H-specific gains overlapped with the regions of the previous study, indicating a worse outcome.

We also assessed these genes with respect to clinical outcomes using the TCGA database. We found that patients with altered copy numbers of defense response genes (*KLRC4*, *CLEC1A, KLRC2*, *ABCC9*, *KLRC3*, *OLR1*, *KCNJ8*, *MS4A2*, *CLEC7A* and *CLEC1B*) had a poorer OS than patients without such alterations. Patients with alterations of immune system and immune cell activation genes (*BLOC1S3*, *KLRK1*, *MS4A2*, *CLEC7A* and *CD5*), as well as cell activation genes (*PLCZ1*, *BLOC1S3*, *MS4A1*, *KLRK1*, *CLEC7A* and *ERCC1*) also had poor OS. However, the differences associated with these three categories of genes were not significant (*P* = 0.245, 0.151, 0.239, respectively). Amplification of nucleotide excision repair, DNA damage removal genes (*DDB1*, *ERCC1* and *ERCC2*) was associated with trends for worse OS (*P* = 0.0662) and PFS (*P* = 0.0919). Both highly invasive/migratory subclones were also characterized by amplification of cell proliferation genes (*BCAT1*, *PRPF19*, *IFLTD1*, *MS4A2*, *CD5*, *ERCC1* and *ERCC2*), which was associated with a significant difference in OS (*P* = 0.0283) but not PFS (*P* = 0.222), and by amplification of genes with products involved in the G1/S transition of the cell cycle (*BCAT1*, *CDKN1B* and *MARK4*), which was associated with a significant difference in OS (*P* = 0.00454) but not PFS (*P* = 0.148) (Supplementary Figure 2–7).

In the analysis of each gene with both A-H- and S-H-specific gains, *TRAPPC6A*, *BLOC1S3*, *EXOC3L2*, *CKM*, KLC3, *ERCC2*, *PPP1R13L*, *CD3EAP*, *ERCC1*, *FOSB, RTN2*, *PPM1N*, *VASP* and *OPA3* amplifications were associated with significantly worse outcomes (OS: all *P* < 0.01; PFS: all *P* < 0.05). *MARK4*, *BCL2L14*, *LRP6*, *MANSC1*, *DUSP16, CREBL2*, *GPR19*, *CDKN1B*, *ST8SIA1* and *PPP1R37* amplifications were associated with significantly differences in OS but not PFS (OS: all *P* < 0.05). *MS4A8*, *MS4A15* and *MS4A10* amplifications were associated with significantly differences in PFS but not OS (PFS: all *P* < 0.05) (Supplementary Table 10).

We then assessed the correlation between copy number and mRNA levels. The altered copy numbers of several genes, including *BLOC1S3*, *ERCC1*, *DDB1*, *ERCC2*, *BCAT1*, *PRPF19*, *CDKN1B*, *MARK4*, *TRAPPC6A*, *KLC3*, *PPP1R13L*, *CD3EAP*, *RTN2*, *VASP*, *OPA3*, *LRP6*, *MANSC1*, *DUSP16*, *CREBL2*, *GPR19* and *CDKN1B*, correlated well with their mRNA levels in the TCGA database (Supplementary Table 10). We also detected the expression levels using RT-PCR and Western blot analysis of all the above genes in our distinct highly and minimally invasive/migratory subclones. We found that the mRNA and protein levels of *DDB1*, *ERCC1*, *ERCC2*, *CKM*, *PRPF19*, *BCAT1*, *PPP1R13L*, *CDKN1B*, *CD3EAP* and *MARK4* were both significantly greater in A-H/S-H cells than in A-L/S-L cells (Supplementary Figure 8).

**Figure 4:**
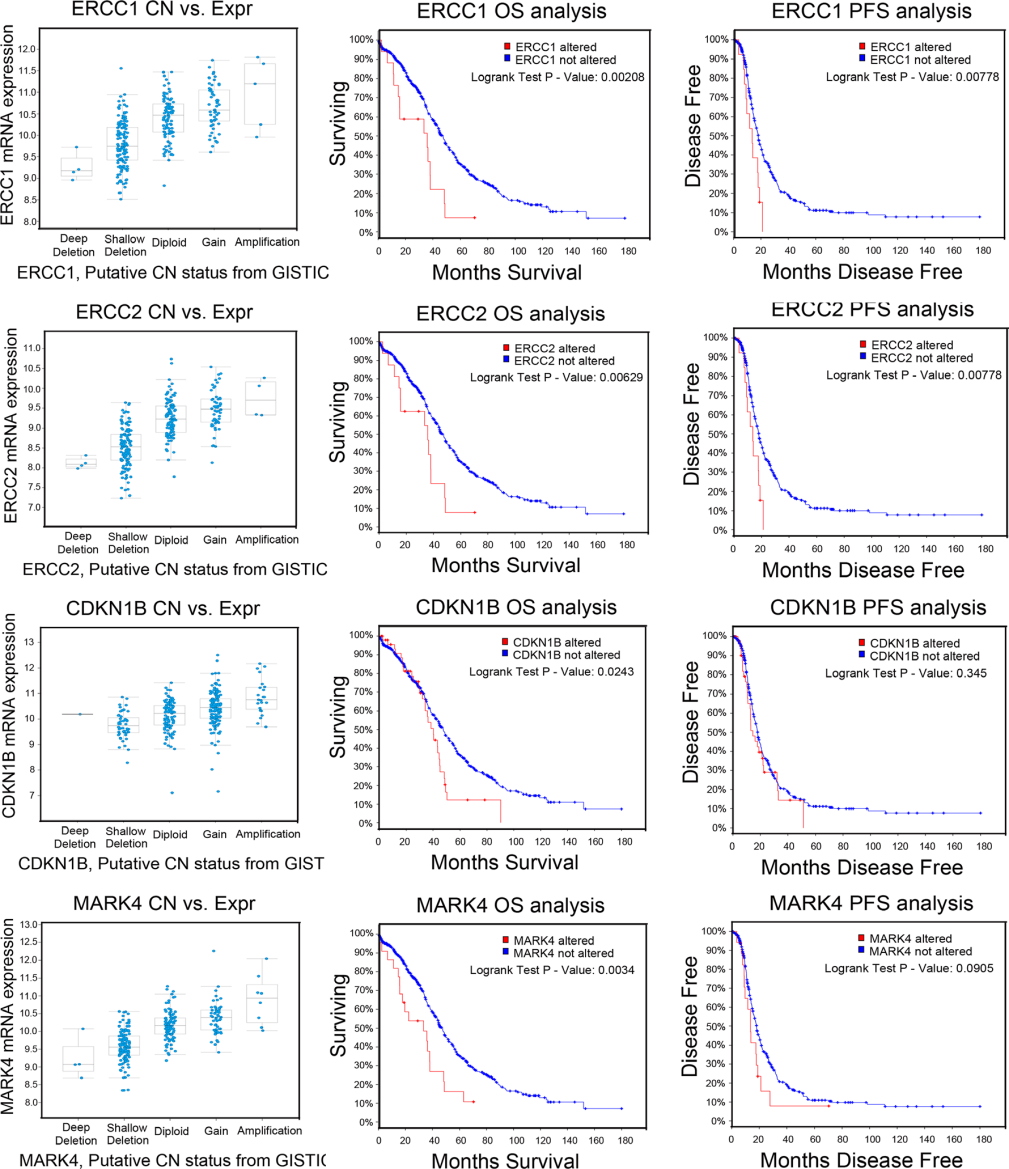
Reproduction of data from the TCGA database with the cBioPortal tool The amplifications of candidate genes, *ERCC1*, *ERCC2*, *CDKN1B* and *MARK4* correlated with gene expression and were associated with patient outcomes. Kaplan-Meier analysis of OS and PFS was performed with the copy number of the candidate gene as a categorical variable, so that the effects of genes with unaltered and altered copy numbers could be compared. The results of a Cox proportional hazards test, with residual disease as a copredictor, are shown as *P* values. The correlations between the copy numbers and the mRNA levels of the candidate genes, determined through RNA Seq analysis of the TCGA datasets, are also shown.

**Figure 5:**
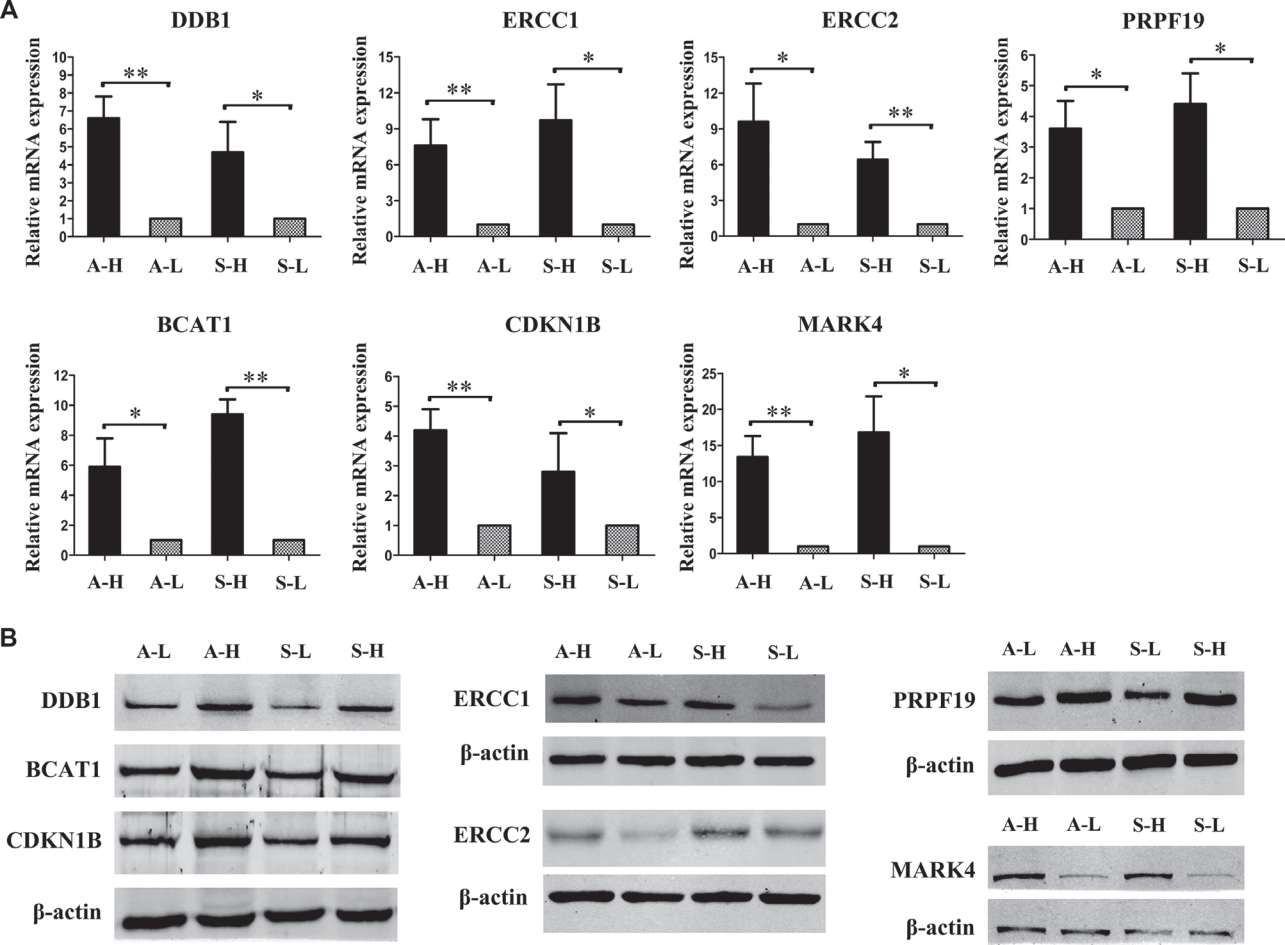
Expression of the candidate genes in highly and minimally invasive/migratory subclones (**A**) RT-PCR and (**B**) Western blot analysis consistently confirmed that *DDB1*, *ERCC1*, *ERCC2*, *PRPF19*, *BCAT1*, *CDKN1B* and *MARK4* expression were all greater in A-H/S-H cells than in A-L/S-L cells. Error bars represent the SEM, *n* = 3 (*P < 0.05. **P < 0.01).

After integrating our analysis of CNV data with gene expression and patient outcome data, we identified 7 genes as strong candidates for therapeutic targeting in advanced ovarian cancer: *DDB1*, *ERCC1*, *ERCC2*, *PRPF19*, *BCAT1*, *CDKN1B* and *MARK4* (Table 1). Relative copy numbers of these genes by quantitative PCR analysis of A-H versus A-L and S-H versus S-L were consistent with the array data (Supplementary Figure 9). Copy number gains correlated well with enhanced expression for *DDB1*, *ERCC1*, *ERCC2*, *PRPF19*, *CDKN1B* and *MARK4*, and with the same trend for *BCAT1*. Elevated *ERCC1* and *ERCC2* levels were consistently associated with worse outcomes (both OS and PFS, *P* < 0.01); *CDKN1B* and *MARK4* amplification and increased expression were associated with significantly worse OS but not PFS (*CDKN1B*, OS: *P* = 0.0243, PFS: *P* = 0.345; *MARK4*, OS: *P* = 0.0034, PFS: *P* = 0.0905) (Figure 4). In addition, COX regression analysis revealed that *ERCC1*, *ERCC2*, *CDKN1B* and *MARK4* were independent prognostic factors for OS. *PRPF19*, *BCAT1* and *DDB1* were identified according to the functional GO groups and their expression levels. Amplification of *PRPF19* was not significantly associated with OS, but rather with a trend for better PFS (*P* = 0.0856). *BCAT1* amplification was not associated with significantly worse outcomes, but was in the functional GO groups of cell proliferation and the G1/S transition of the cell cycle. Elevated *DDB1* was not associated significantly with survival, but belonged to the group of DNA damage removal genes (Supplementary Figure 10). RT-PCR and Western blot analyses of our highly and minimally invasive/migratory subclones consistently confirmed the expression levels of the seven genes (Figure 5).

**Table 1:** Analyses of candidate genes in the TCGA ovarian cancer database (TCGA, Provisional) with the cBioPortal tool

Genes	Location	Correlation between copy number and gene expression level		Survival (*P* value)	
		Pearson	Spearman	OS	PFS
*DDB1*	11q12.2	0.565	0.523	0.89	0.961
***ERCC1***	19q13.32	0.547	0.626	**0.00208**	**0.00778**
***ERCC2***	19q13.32	0.63	0.696	**0.00629**	**0.00778**
*PRPF19*	11q12.2	0.478	0.461	0.451	0.0856
***CDKN1B***	12p13.1	0.492	0.452	**0.0243**	0.345
*BCAT1*	12p12.1	0.42	0.262	0.152	0.729
***MARK4***	19q13.32	0.699	0.717	**0.0034**	0.0905

## DISCUSSION

Studies performed by others [30–32] and in our lab [33–35] have demonstrated that there is heterogeneity among ovarian cancers. We used the limiting-dilution method to isolate and establish heterogeneous subclones of the ovarian cancer cell lines A2780 and SKOV3 [22]. Among these subclones A-H/S-H and A-L/S-L cells exhibited the highest and lowest invasive/migratory capacities, respectively. A-H and S-H cells displayed enhanced proliferation and anti-apoptotic activity, as well as significant resistance to cisplatin and Taxol *in vitro*, and a higher capacity for tumor formation *in vivo*, compared with A-L and S-L cells, respectively. These studies demonstrated that we successfully produced a model consisting of clones with distinct invasive/migratory capacities and the same hereditary background. We hypothesized that heterogeneity may preferentially evolve due to initial alterations in DNA, followed by alterations in chromosomal copy numbers. In the present study, we used array-based technology to systematically analyze CNVs in the heterogeneous invasive/migratory models. We also integrated our data with clinical findings to determine the relationship among CNVs, gene expression and patient outcomes, and thus identified a number of genes that may be good therapeutic targets.

Several studies have described the copy number aberrance in ovarian cancer, and our data agreed well with these previous CNV analyses. However, it was clear that the A2780-derived subclones differed extensively from the SKOV3-derived subclones. This may have contributed to cell-line-specific characteristics and the heterogeneity of these ovarian cancer cell lines. The A2780 cell line is probably of endometrioid origin, while the SKOV3 cell line is of clear cell carcinoma origin [36]. The different ovarian cancer histotypes practically represent genetic disparities. Moreover, such heterogeneity could also be due to the cellular phenotype differences between the two cell lines. A2780 cells exhibit an epithelial/intermediate (round) phenotype accompanied with robust expression of epithelial markers, while SKOV3 cells display a typical mesenchymal (spindle-like) morphology accompanied with predominant expression of mesenchymal markers [37]. Accordingly, some of the differences observed in the CNV patterns between the two cell lines might be cellular phenotype-related. In this sense in our results, for example, the A-H-specific loss genes were significantly enriched in cell adhesion and extracellular structure, which cold promote epithelial-to-mesenchymal transition, indicating a mesenchymal-like change in A-H cells.

We performed functional enrichment analysis of our data set to identify genes of specific annotations that were distinctively altered in A-H and A-L, S-H and S-L pairs. It is possible that different mechanisms were responsible for the two distinct cell lines. We observed that “A-H-specific gains” were enriched in signal transduction through G-protein coupled receptors, which involve in many biological functions, such as the sensing of taste, light and odor, chemotaxis, and inflammatory and immune responses [38, 39]. In addition, the loss of genes involved in cell-cell adhesion in A-H cells might have contributed to their high level of invasion/migration. On the other hand, the “S-H-specific gains” were distinctly enriched in microtubule polymerization and vasodilation, as well as regulation of growth and negative regulation of apoptosis/programmed cell death/cell adhesion, which may have promoted cell proliferation and metastasis. These findings strongly suggest that distinct genetic pathways caused the distinct invasive/migratory capacities of the A2780- and SKOV3-derived subclones.

It was also clear from our analysis that some of the same pathways promoted the higher invasive/migratory capacities of A-H and S-H cells, including defense responses, nucleotide excision repair, DNA damage removal, positive regulation of immune system processes, cell activation, cell proliferation and the G1/S transition of the cell cycle. An immune signature has also been shown to be involved in pathological inflammatory conditions and cancer [40]. The expression of inflammation-related programs due to the activation of oncogenes induces the formation of an inflammatory microenvironment, which in turn promotes carcinogenesis [41]. The enrichment of the GO categories of nucleotide excision repair, DNA damage removal and cell proliferation was also consistent with the functional attributes of the A-H and S-H subclones. In contrast, A-L and S-L both had amplicons enriched mainly in cell apoptosis and death, demonstrating a relationship between clonal genomic changes and the low invasive/migratory capacities.

GO analysis allowed us to group the affected genes according to their biological processes, providing new insights into tumor metastasis. Our proof-of-principle screen indicated that several genes had strong prognostic significance for advanced ovarian tumors. A previous study indicated that the expression of immune response genes was elevated and survival was significantly lower in patients with high-grade serous and endometrioid tumors than in those with low-grade/low-malignant-potential tumors [29]. While the amplification of groups of defense response genes in the TCGA database, as well as immune system and immune cell activation genes, was associated with worse OS in our study, the differences were not significant. Immune responses have long been considered important events in tumor progression. Thus, it is necessary to further explore the behavior of ovarian cancer in the context of immune responses.

The amplification of the DNA damage repair genes *DDB1*, *ERCC1* and *ERCC2* was associated with worse outcomes in our study. DDB1, encoded by a gene located at chromosome 11q12.2, is an important positive regulator of nucleotide excision repair [42] and is responsible for resistance to platinum-based agents [43]. ERCC1/2, which are encoded by genes located at chromosome 19q13.32, are the two major components of the nucleotide excision repair process, particularly for DNA damage caused by chemotherapeutic agents [44, 45]. Studies have suggested the use of *ERCC1/2* as molecular predictors of clinical resistance to platinum-based chemotherapy [46]. These conclusions may help to explain our previous data showing that A-H/S-H cells exhibited greater resistance to cisplatin and Taxol than A-L/S-L cells. In addition, our data revealed that *ERCC1/2* amplification was associated with significantly worse OS and PFS. These results indicate that *DDB1*, *ERCC1* and *ERCC2* could be used as predictors of chemoresistance, and that *ERCC1/2* could predict poor clinical outcomes.

Besides immune signature and DNA damage repair genes, genes encoding proteins involved in cell proliferation and G1/S transition were notable for their association with worse OS. Among the genes herein, *PRPF19*, *BCAT1*, *CDKN1B*, and *MARK4* could be considered as potential candidates for therapeutic intervention. PRPF19 (pre-mRNA-processing factor 19) is a U-box-containing E3 ubiquitin ligase and is involved in the DNA damage response [47]. *PRPF19* expression was found to be greater in gastric cancer tissues and/or metastatic lymph nodes than in peri-cancerous tissues [48]. Another report indicated that PRPF19 expression was elevated in most hepatocellular carcinoma tissues and cell lines, and its overexpression correlated positively with vascular invasion and tumor capsule breakthrough, probably through the p38 mitogen-activated protein kinase/twist1 pathway [49]. To date, PRPF19 has not been studied in ovarian cancer; however, these previous studies imply that the gain of *PRPF19* is a critical event during the progression of cancer, making it a promising target for malignancies with aberrant *PRPF19* expression.

*BCAT1* is highly overexpressed in serous ovarian cancers [50]. Knockdown of *BCAT1* dramatically reduced the rates of cell proliferation, migration and invasion, and silencing of *BCAT1* was reported to suppress ovarian tumorigenesis and induce the expression of several tumor suppressors. Additionally, survival was prolonged when *BCAT1* was suppressed in a xenograft model of advanced epithelial ovarian cancer [51]. Thus, this transaminase could be considered a novel malignancy biomarker and a putative therapeutic target.

p27 protein is encoded by the cyclin-dependent kinase inhibitor 1B (*CDKN1B*) gene, mapped to chromosome 12p13. Polymorphisms in *CDKN1B* may be associated with reduced susceptibility to cancer, particularly ovarian cancer [52]. In a global online biomarker validation platform developed to mine all available microarray data in 1287 ovarian cancer patients, *CDKN1B* expression was found to be associated with survival [53]. We also detected amplification and overexpression of *CDKN1B* in both A-H and S-H cells; thus, it will be necessary to examine the pathological processes of *CDKN1B* CNV in advanced-stage ovarian cancer.

The *MARK4* gene encodes a member of the microtubule affinity-regulating kinase family, and is inseparably linked with many human diseases, including cancer, diet-induced obesity, type 2 diabetes, and neurodegenerative disorders. MARK4 was found to bind with a number of proteins linked to cell motility, clearly suggesting its involvement in the promotion of the cytoskeleton. It is also involved in the stimulation of cell migration and metastasis in breast and lung cancers [54–56]. To date, MARK4 has been found to contribute to the development of hepatocellular carcinomas [57], gliomas [58], prostate cancer [59] and breast cancer [60]. However, no research on MARK4 has been performed for ovarian cancer, so further study is required regarding its potential involvement in the metastasis of ovarian carcinomas.

In summary, we have systematically analyzed the functions of the altered genes in ovarian cancer cell line models with heterogeneous invasive/migratory capacities. Integrating the data on copy number diversity with clinical outcomes and mRNA/protein expression facilitated the search for potential therapeutic targets. Therefore, we predict that clone-specific functional and genetic profiling will be a helpful method of identifying new molecular pathways underlying cancer and new biomarkers for clinical applications. This will not only reveal the heterogeneity in ovarian cancers of different developmental stages, but also elucidate other factors that account for cancer deaths, such as genes that promote chemoresistance.

## MATERIALS AND METHODS

### Cell culture

The human ovarian cancer cell lines A2780 and SKOV3 were purchased from the Cell Support Center, Institute of Basic Medical Science, Chinese Academy of Medical Sciences. A2780 was established from ovarian tumor tissue obtained from an untreated patient in the UK [61, 62]. SKOV3 cells, which were isolated from the ascites of a patient with ovarian adenocarcinoma, are resistant to several cytotoxic drugs, including diphtheria toxin, cis-platinum and Adriamycin [63]. A2780 and SKOV3 are the most popular cell line models for ovarian cancer studies in the literature. Their highly and minimally invasive/migratory subclones were cultured in Roswell Park Memorial Institute (RPMI)-1640 medium (Hyclone, Logan, Utah, USA) supplemented with 10% heat-inactivated FBS (Gibco, Carlsbad, CA, USA), 100 μg/mL streptomycin and 100 IU/mL penicillin at 37°C in a humid incubator containing 5% CO_2_. The cells were subcultured when they reached approximately 80% confluence.

### CNV assay

Genomic DNA was extracted from the cultured cells with a Gentra Puregene kit (Qiagen, Hilden, Germany). The DNA samples were genotyped with a CytoScan™ HD array (Affymetrix, Santa Clara, CA, USA) according to the manufacturer's instructions. This array contains 2,696,550 CNV markers, including 743,304 genotypable SNP probes and 1,953,246 non-polymorphic probes. The data were visualized and then analyzed in the Affymetrix Chromosome Analysis Suite (ChAS) software package [64].

### Validation of gene CNVs

We selected a subset of regions identified as varying in copy number between A-H and A-L, S-H and S-L. Genomic DNA was extracted from cultured cells with a Gentra Puregene kit (Qiagen) according to the manufacturer's instructions. The primers listed in Supplementary Table 1 were used to determine the relative gene copy numbers between A-H and A-L, S-H and S-L. PCR was conducted on a CFX 96 Real-Time PCR Detection System (Bio-Rad Laboratories, Hercules, CA, USA) with a SYBR Green PCR Kit (Takara Bio Inc., Otsu, Japan). The following PCR protocol was used: 95°C for 10 min, followed by 40 cycles of amplification at 95°C for 15 s and 60°C for 1 min. Dissociation curve analyses were conducted to confirm the specificity of the PCR products. The gene copy numbers were normalized against the levels of RNase P-2 and analyzed by the comparative Ct method (ΔΔCt). The mean values of the fold changes obtained in three independent experiments were calculated.

### Gene copy numbers in ovarian cancer samples and clinical outcomes

We obtained copy number data for 603 high-grade serous ovarian cancer samples from The Cancer Genome Atlas (TCGA) [65]. The data in the TCGA ovarian serous cystadenocarcinoma database were analyzed with the cBioPortal online analytical tool (http://www.cbioportal.org/public-portal/) in the “TCGA, provisional” category. The clinical copy number profiles were summarized through “OncoPrint.” The “Plot” function was used to generate copy number status/mRNA expression (RNA Seq V2 RSEM) (log2) correlation plots. The gene expression values were compared with matching copy number values by means of Spearman and Pearson correlations. The “Survival” function was used to plot Kaplan-Meier curves [66, 67]. Multivariate analysis for independent prognostic factors was performed using COX regression model.

### RNA isolation and real-time PCR assay for mRNA expression

Total RNA was extracted with the TRIzol reagent (Invitrogen, Carlsbad, CA, USA) according to the manufacturer's instructions. cDNA was then synthesized with a QuantScript Reverse Transcription Kit (Tiangen, China). The primers are listed in Supplementary Table 2. Real-time PCR was performed on a CFX 96 Real-Time PCR Detection System with a SYBR Green PCR Kit. The analysis of each sample was repeated at least three times.

### Western blot

Cells were prepared in cold radioimmunoprecipitation assay lysis buffer (Applygen technology, China) containing freshly added 0.01% protease inhibitor (Sigma, Louis, MO, USA) for 30 min. The solutions were centrifuged at 12000 × *g* for 10 min at 4°C, and the supernatants were collected. Approximately 50 μg of total protein was separated on a 10–15% SDS-polyacrylamide gel and transferred to a nitrocellulose membrane. After being blocked with 5% non-fat milk in Tris-buffered saline containing 0.1% Tween-20 for 2 h at room temperature, the membranes were incubated with primary antibodies overnight at 4°C, followed by horseradish peroxidase-conjugated secondary antibodies (Santa Cruz, CA, USA) for 2 h at room temperature. The signals were visualized with a SuperEnhanced Chemiluminescence Detection Kit (Applygen technology, China). Details about the primary antibodies are presented in Supplementary Table 3. Each assay was performed at least three times.

### Statistical analysis

Statistical analysis of the data was performed with SPSS 17.0 software (SPSS, Chicago, Illinois, USA). The data were expressed as the mean values ± standard errors of the mean. The significance of differences in values was evaluated through analysis of variance or an unpaired two-tailed Student's *t-test*. A *P value* < 0.05 was considered to indicate a significant difference. All experiments were repeated at least three times.

## SUPPLEMENTARY MATERIALS FIGURES AND TABLES
















