# Supplementary material for: Genome-wide DNA copy number analysis in clonally expanded human ovarian cancer cells with distinct invasive/migratory capacities
Source: Oncotarget. 2017 Jan 20;8(9):15136–48. doi: 10.18632/oncotarget.14767 (PMC5362473; doi:10.18632/oncotarget.14767)
Supplement: Supplementary file 1 [file oncotarget-08-15136-s001.pdf]

Genome-wide DNA copy number analysis in clonally expanded human ovarian cancer cells with distinct invasive/migratory capacities

Supplementary Materials

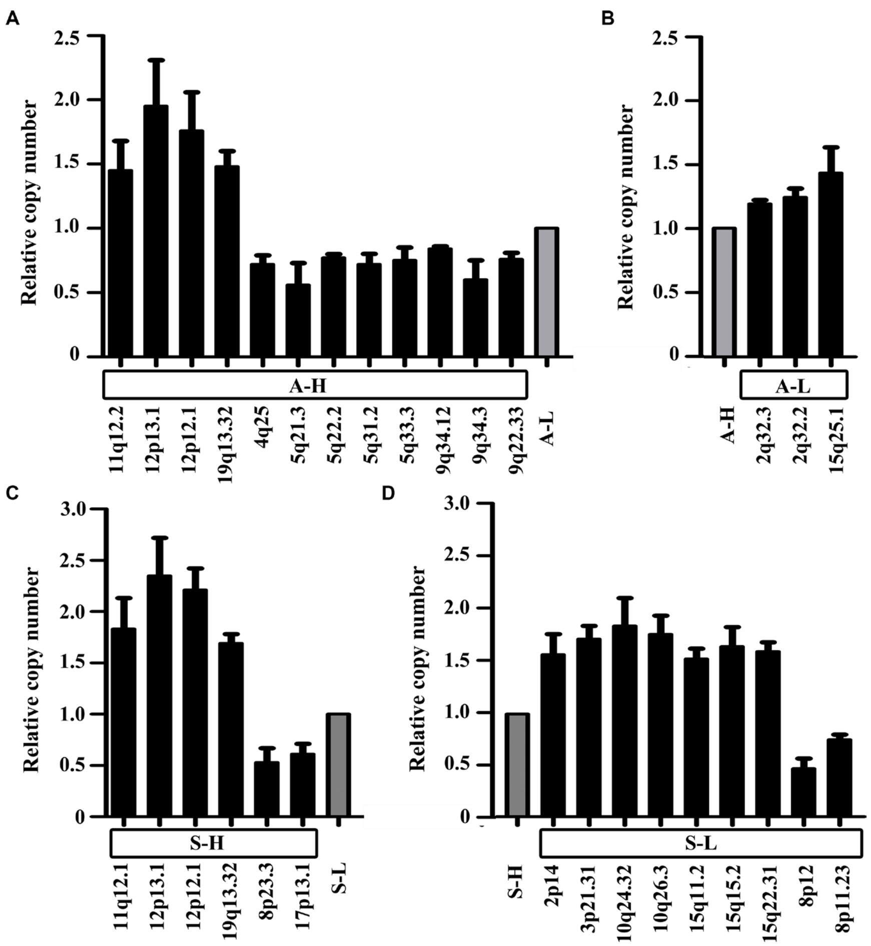

Supplementary Figure 1: Validation of the CNV data. Relative gene copy numbers in the selected regions were determined by RT-PCR with DNA from (**A**, **B**) A-H and A-L or (**C**, **D**) S-H and S-L cells. The data shown are the average values from three independent experiments ± SEM. For (A,C), the relative gene copy numbers of A-L/S-L cells were set as 1 and those of A-H/S-H cells were normalized to these values, whereas for (B,D), the relative gene copy numbers of A-H/S-H cells were set as 1 and those of A-L/S-L cells were normalized to these values (For each relative copy number of each selected region, $P < 0.05$; data not shown.) .

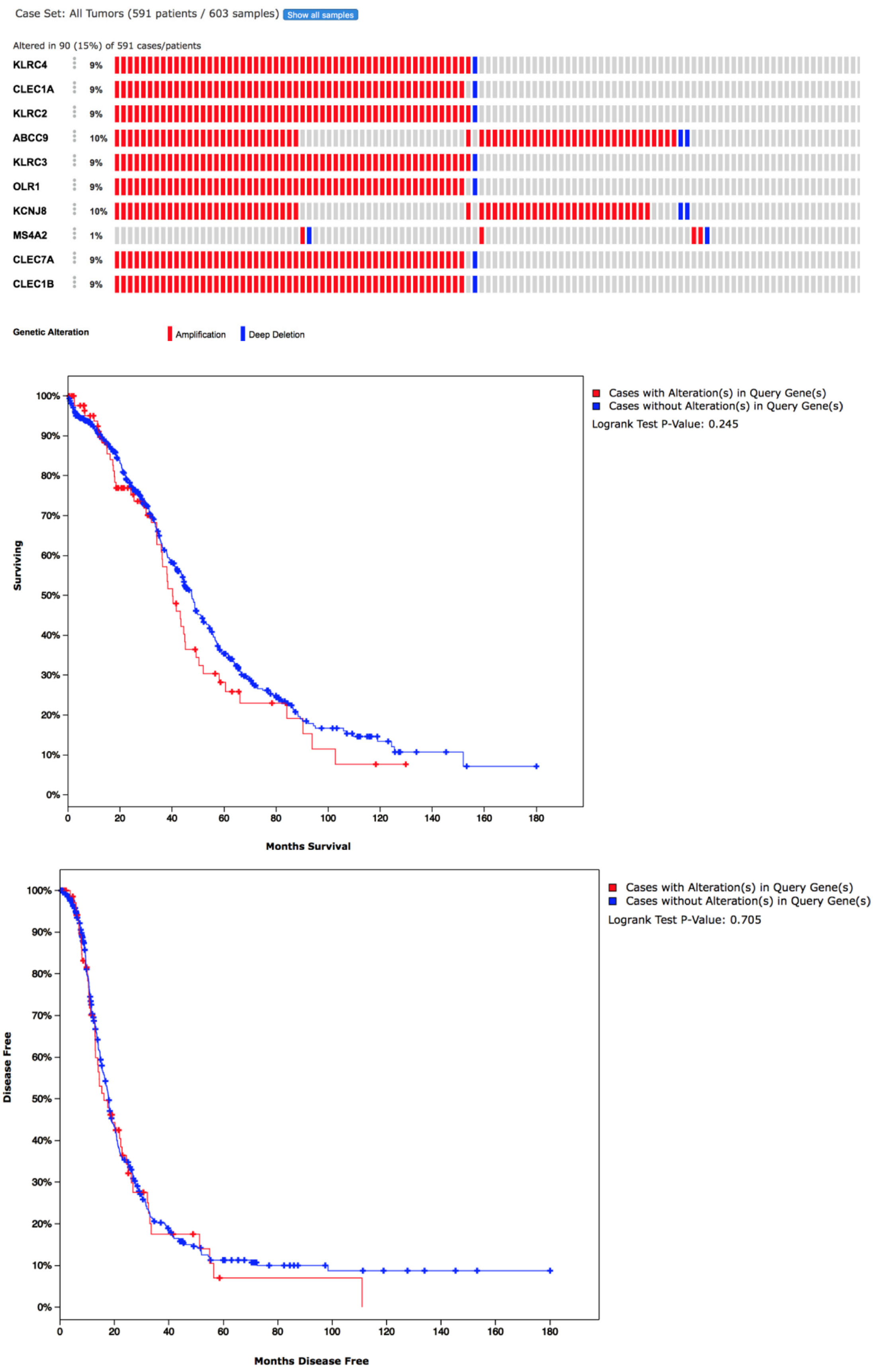

Supplementary Figure 2: Clinical outcomes associated with the functional gene groups amplified in both A-H- and S-H-specific gains from the TCGA database. For each gene group, copy number alterations of the member genes are listed. Kaplan-Meier analysis of OS and PFS was performed with the copy number of the gene group as a categorical variable, so that the groups with unaltered and altered copy numbers could be compared. The results of a Cox proportional hazards test, with residual disease as a copredictor, are shown as *P* values. The diagram depicts the detailed number of patients and survival months with unaltered and altered copy numbers.

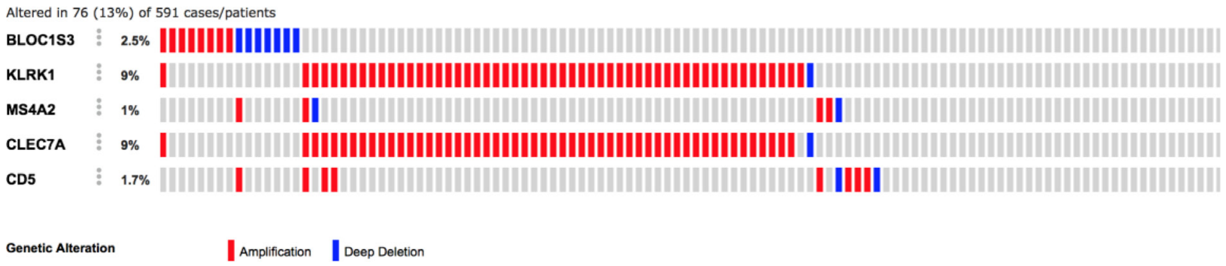

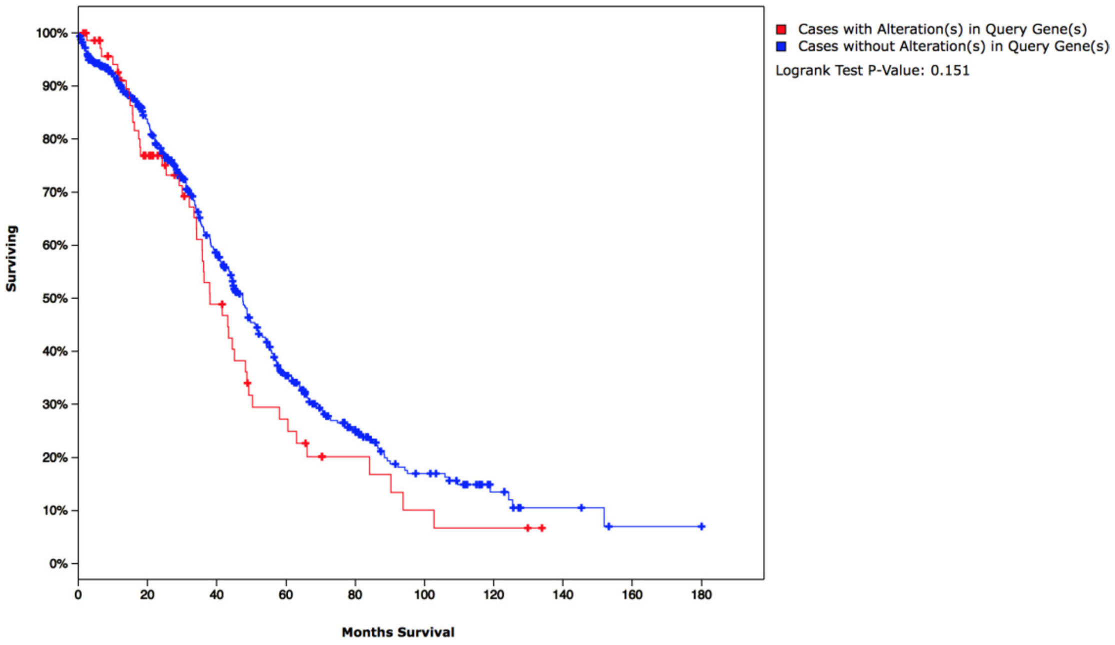

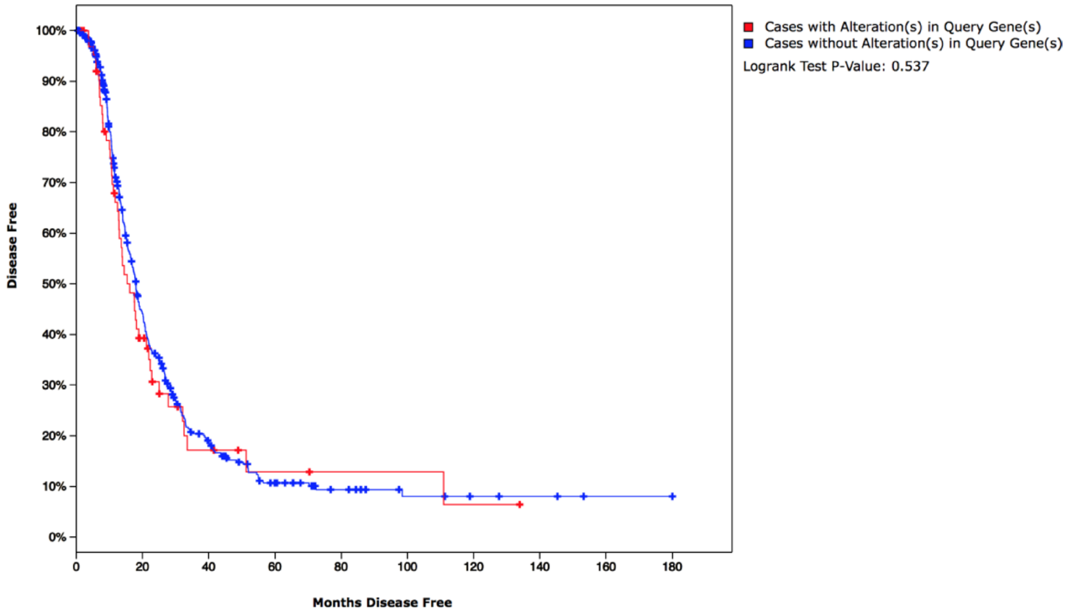

Supplementary Figure 3: Clinical outcomes associated with the functional gene groups amplified in both A-H- and S-H-specific gains from the TCGA database. For each gene group, copy number alterations of the member genes are listed. Kaplan-Meier analysis of OS and PFS was performed with the copy number of the gene group as a categorical variable, so that the groups with unaltered and altered copy numbers could be compared. The results of a Cox proportional hazards test, with residual disease as a copredictor, are shown as *P* values. The diagram depicts the detailed number of patients and survival months with unaltered and altered copy numbers.

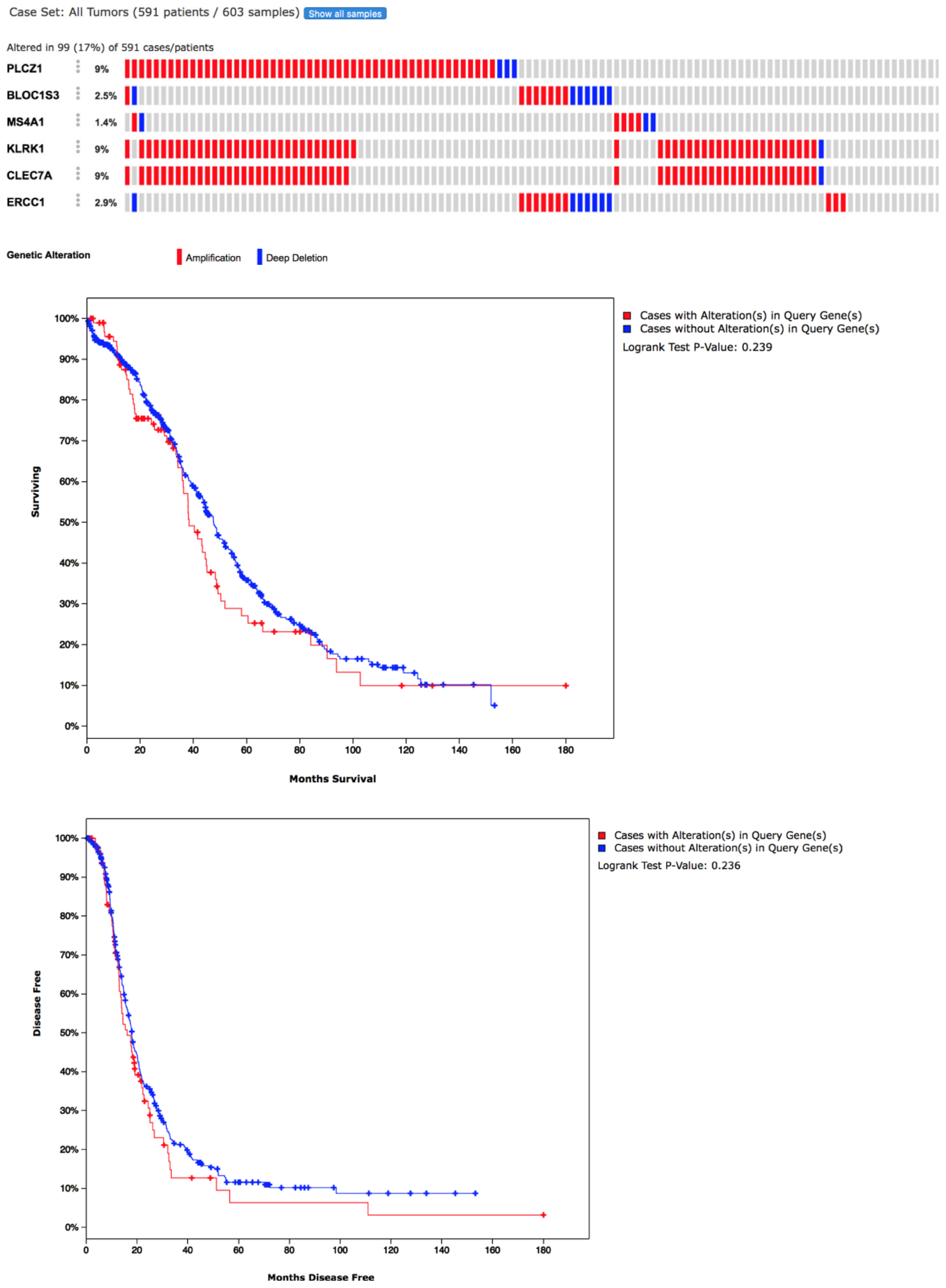

Supplementary Figure 4: Clinical outcomes associated with the functional gene groups amplified in both A-H- and S-H-specific gains from the TCGA database. For each gene group, copy number alterations of the member genes are listed. Kaplan-Meier analysis of OS and PFS was performed with the copy number of the gene group as a categorical variable, so that the groups with unaltered and altered copy numbers could be compared. The results of a Cox proportional hazards test, with residual disease as a copredictor, are shown as *P* values. The diagram depicts the detailed number of patients and survival months with unaltered and altered copy numbers.

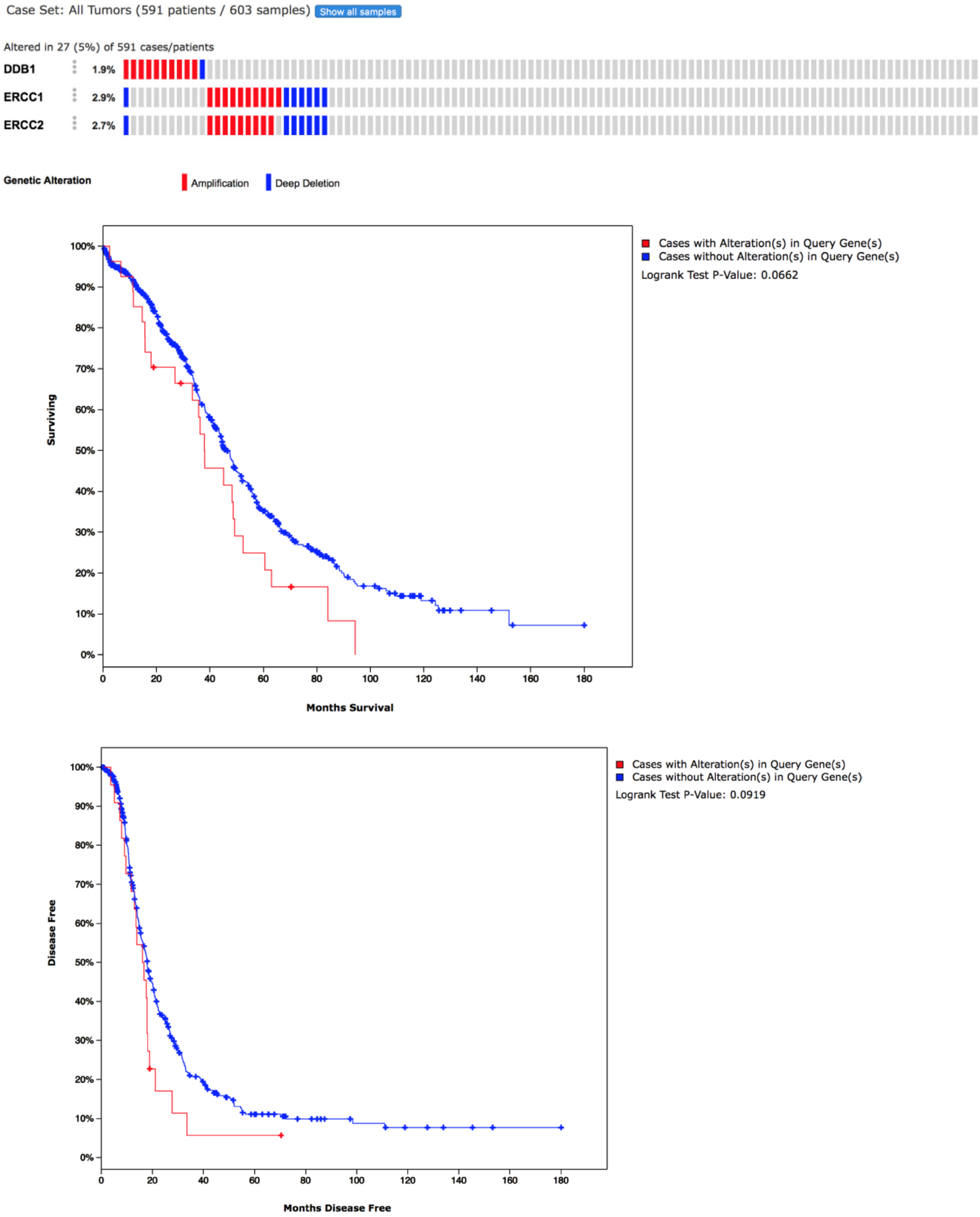

Supplementary Figure 5: Clinical outcomes associated with the functional gene groups amplified in both A-H- and S-H-specific gains from the TCGA database. For each gene group, copy number alterations of the member genes are listed. Kaplan-Meier analysis of OS and PFS was performed with the copy number of the gene group as a categorical variable, so that the groups with unaltered and altered copy numbers could be compared. The results of a Cox proportional hazards test, with residual disease as a copredictor, are shown as *P* values. The diagram depicts the detailed number of patients and survival months with unaltered and altered copy numbers.

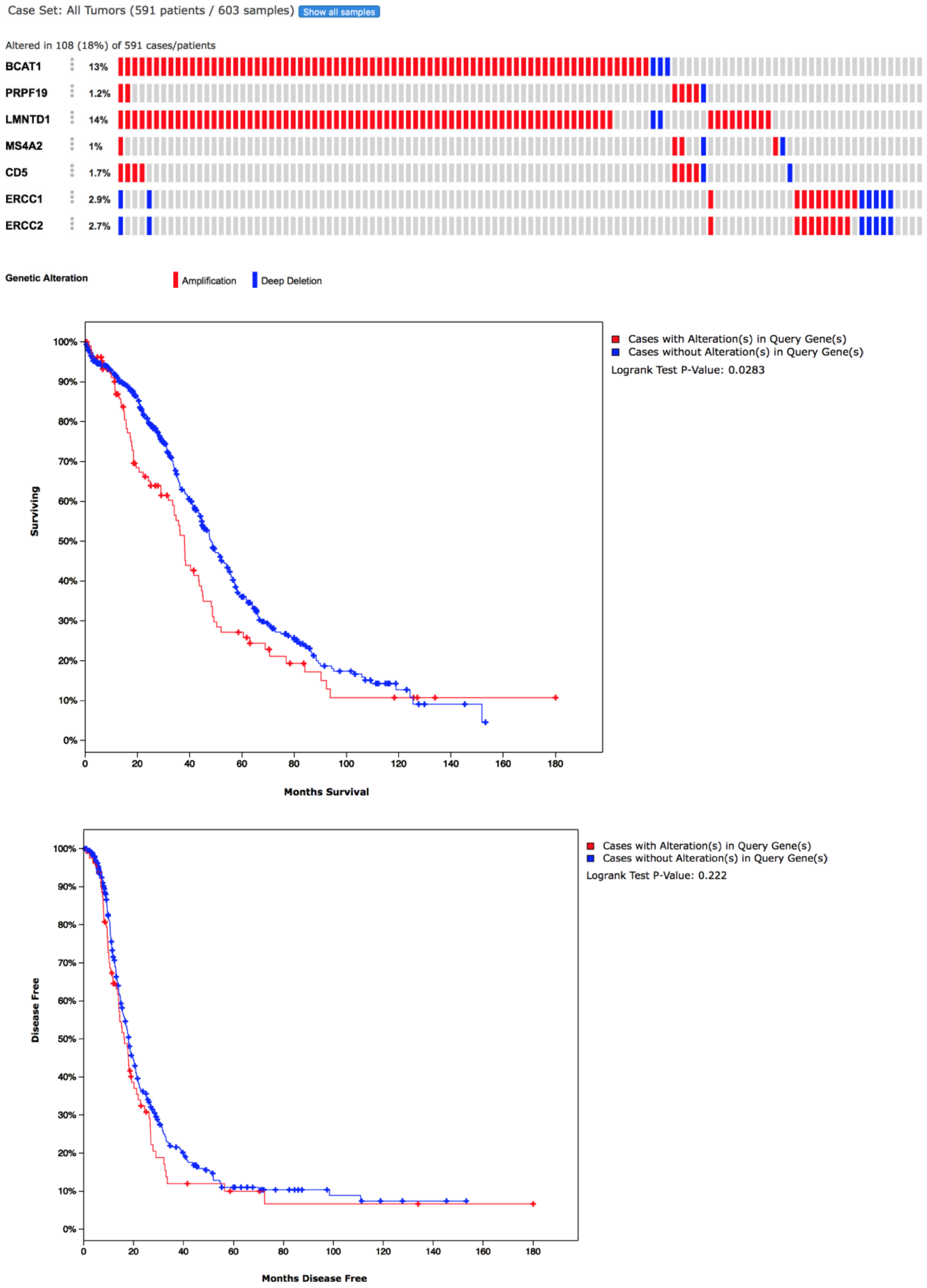

Supplementary Figure 6: Clinical outcomes associated with the functional gene groups amplified in both A-H- and S-H-specific gains from the TCGA database. For each gene group, copy number alterations of the member genes are listed. Kaplan-Meier analysis of OS and PFS was performed with the copy number of the gene group as a categorical variable, so that the groups with unaltered and altered copy numbers could be compared. The results of a Cox proportional hazards test, with residual disease as a copredictor, are shown as *P* values. The diagram depicts the detailed number of patients and survival months with unaltered and altered copy numbers.

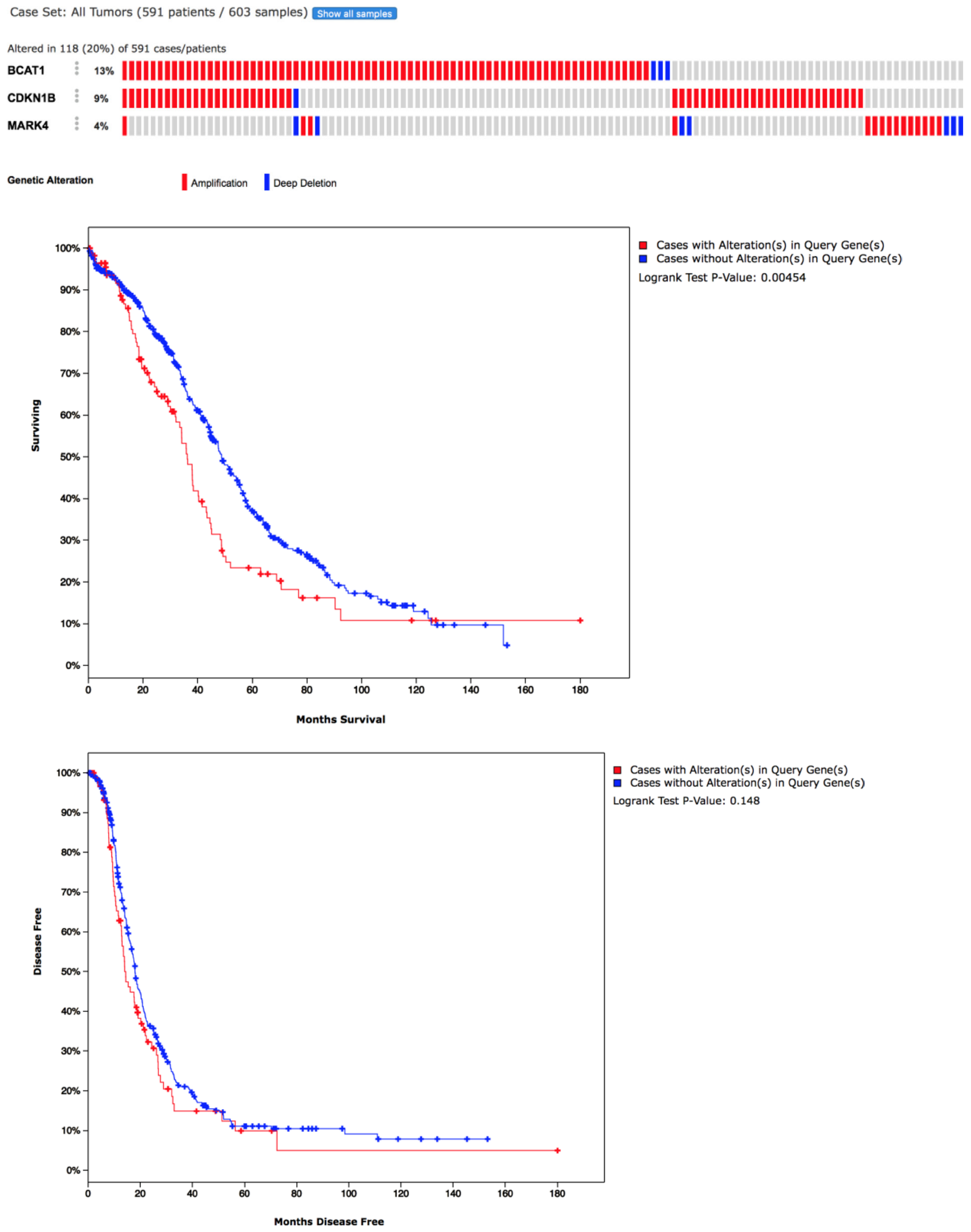

Supplementary Figure 7: Clinical outcomes associated with the functional gene groups amplified in both A-H- and S-H-specific gains from the TCGA database. For each gene group, copy number alterations of the member genes are listed. Kaplan-Meier analysis of OS and PFS was performed with the copy number of the gene group as a categorical variable, so that the groups with unaltered and altered copy numbers could be compared. The results of a Cox proportional hazards test, with residual disease as a copredictor, are shown as *P* values. The diagram depicts the detailed number of patients and survival months with unaltered and altered copy numbers.

Genes	Differential expression		*P* value	
	**A-H VS A-L**	**S-H VS S-L**	**A-H VS A-L**	**S-H VS S-L**
MS4A2	--	--	ns	ns
CD5	--	--	ns	ns
ERCC1	↗	↗	**	*
DDB1	↗	↗	**	*
ERCC2	↗	↗	*	**
BCAT1	↗	↗	*	**
PRPF19	↗	↗	*	*
IFLTD1	--	--	ns	ns
CDKN1B	↗	↗	**	*
MARK4	↗	↗	**	*
TRAPPC6A	↗	--	**	ns
EXOC3L2	↗	--	***	ns
CKM	↗	↗	*	*
KLC3	↘	↗	ns	ns
PPP1R13L	↗	↗	*	*
CD3EAP	↗	↗	**	**
FOSB	↗	↘	*	*
RTN2	↗	↗	*	*
PPM1N	↘	↘	*	ns
VASP	↗	--	*	ns
OPA3	↗	↗	*	*
BCL2L14	↘	--	ns	ns
LRP6	↗	↗	*	*
MANSC1	↗	--	ns	ns
DUSP16	↗	↗	**	ns
CREBL2	--	↗	ns	*
GPR19	↗	↘	*	**
ST8SIA1	↗	--	*	ns
PPP1R37	↘	--	*	ns
MS4A8	--	--	ns	ns
MS4A15	--	--	ns	ns

Note: ↗: increase; ↘: decrease; --: no change; *: $P < 0.05$; **: $P < 0.01$; ***: $P < 0.001$; ns: not significant.

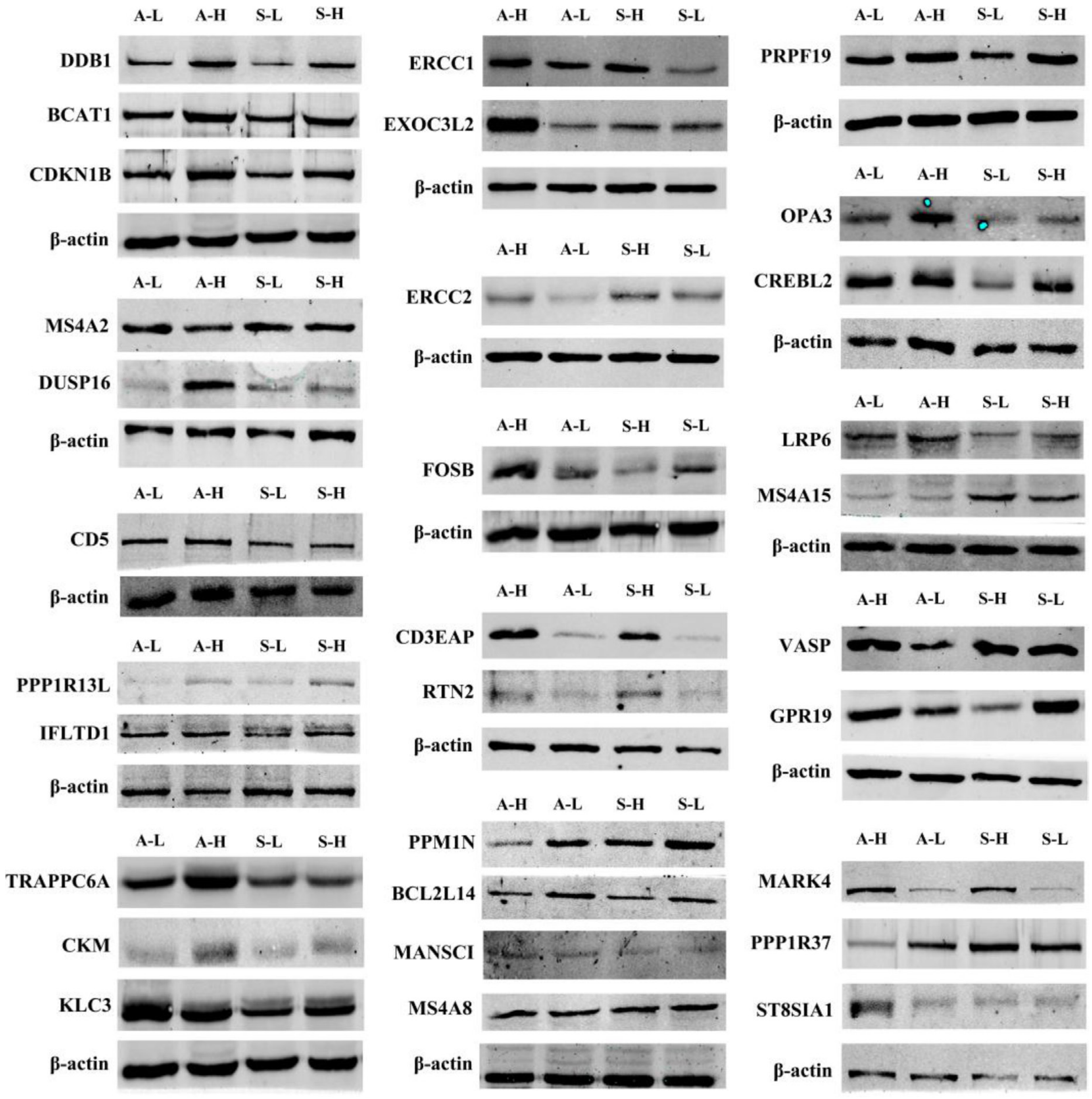

A-L A-H S-L S-H
DDB1
BCAT1
CDKN1B
β-actin
A-L A-H S-L S-H
MS4A2
DUSP16
β-actin
A-L A-H S-L S-H
CD5
β-actin
A-L A-H S-L S-H
PPP1R13L
IFLTD1
β-actin
A-L A-H S-L S-H
TRAPPC6A
CKM
KLC3
β-actin
A-H A-L S-H S-L
ERCC1
EXOC3L2
β-actin
A-H A-L S-H S-L
ERCC2
β-actin
A-H A-L S-H S-L
FOSB
β-actin
A-H A-L S-H S-L
CD3EAP
RTN2
β-actin
A-H A-L S-H S-L
PPM1N
BCL2L14
MANSCI
MS4A8
β-actin
A-L A-H S-L S-H
PRPF19
β-actin
A-L A-H S-L S-H
OPA3
CREBL2
β-actin
A-L A-H S-L S-H
LRP6
MS4A15
β-actin
A-H A-L S-H S-L
VASP
GPR19
β-actin
A-H A-L S-H S-L
MARK4
PPP1R37
ST8SIA1
β-actin

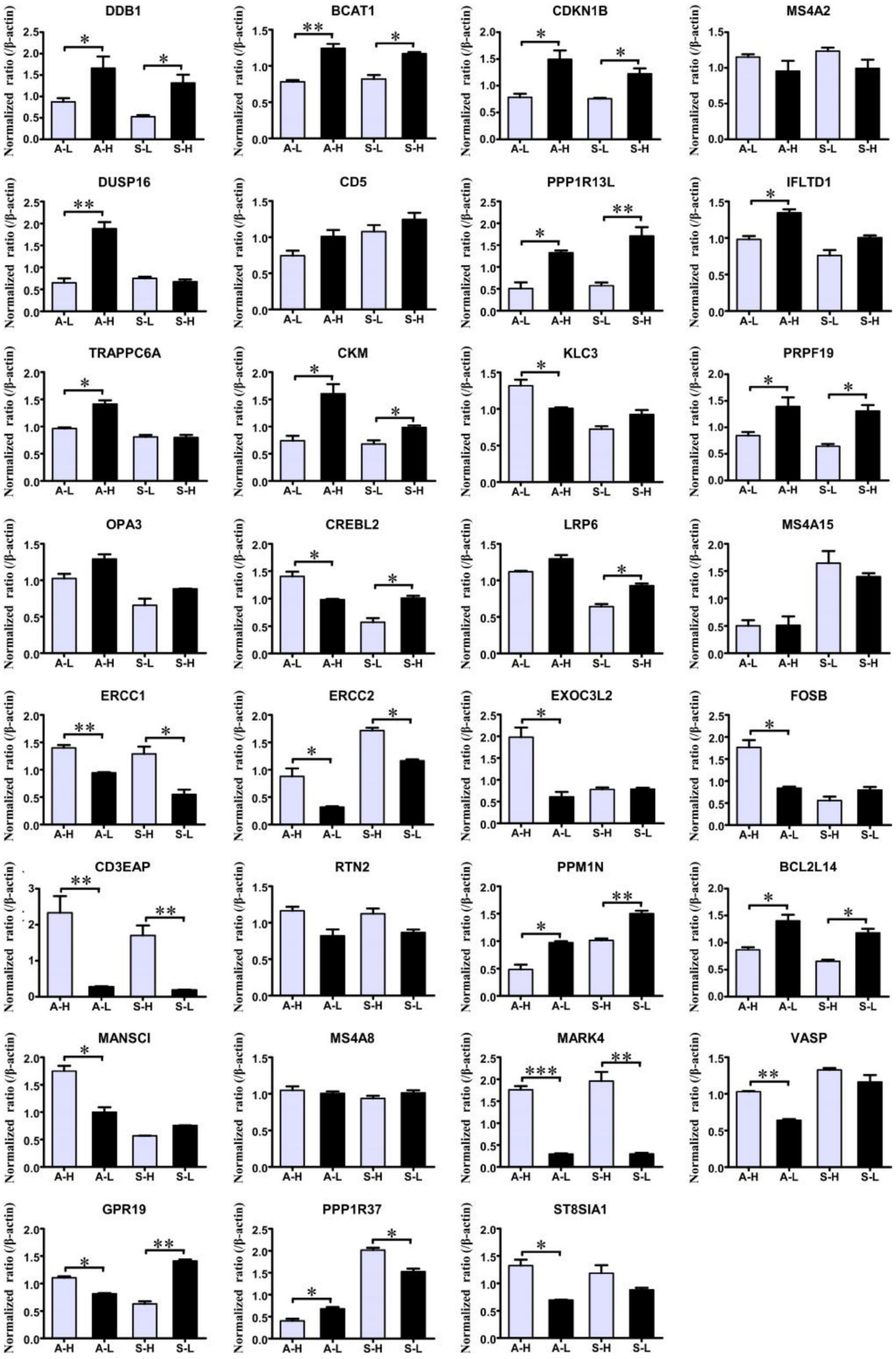

Supplementary Figure S8: Expression levels of all the selected genes in the distinct invasive/migratory subclones. (**A**) RT-PCR analysis of the mRNA expression of the selected genes. (**B**) Western blot analysis displayed the protein levels of all the selected genes in A-H vs. A-L and S-H vs. S-L. (**C**) The data in the bar graphs were normalized to β-actin. Error bars represent the SEM, $n = 3$ ($^*P < 0.05$. $^{**}P < 0.01$. $^{***}P < 0.001$).

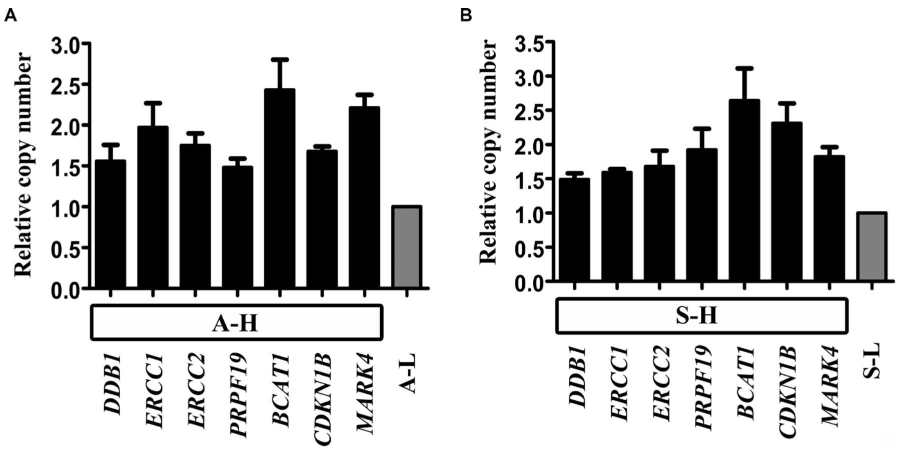

Supplementary Figure 9: Validation of the CNV data of the candidate genes. Relative gene copy numbers of the 7 candidate genes were determined by RT-PCR with DNA from (**A**) A-H and A-L or (**B**) S-H and S-L cells. The data shown are the average values from three independent experiments ± SEM. The relative gene copy numbers of A-L/S-L cells were set as 1 and those of A-H/S-H cells were normalized to these values (For each relative copy number of each gene, $P < 0.05$; data not shown).

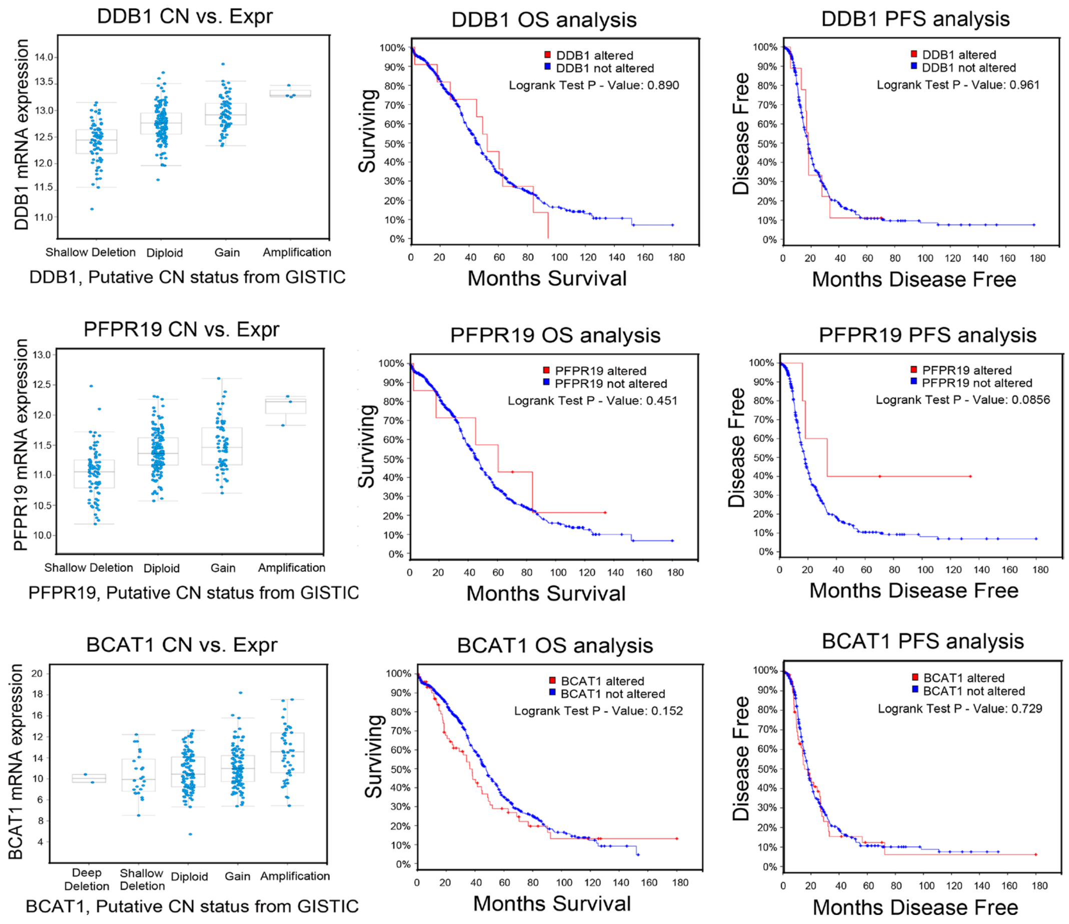

Supplementary Figure 10: Reproduction of data from the TCGA database with the cBioPortal tool. The graphs indicated the association of amplifications of candidate genes *DDB1*, *PFPR19* and *BCAT1* with gene expression and patient outcomes.

Supplementary Table 1: Primers used for qPCR validation of CNVs

Primer name	Forward	Reverse	Amplicon/bp
RNase P-2	TTTTACTCCCACAGCACCTCC	GGAATTTGGAGTGACCAAAGG	105
**A-H-specific gain**			
11q12.2	AAAAGCGCTCCATCTCCCAA	CTGGGTTGGGACAGAGTCAA	140
12p13.1	CAGCTTGCCCGAGTTCTACT	TGTCCTCAGAGTTAGCCGGA	137
12p12.1	CACAAAGAAAGCCCTCCCCA	CTGTGTTTCTCCCTTCTCAGGAT	150
19q13.32	GTCGCCAGAAATGGATGTGC	CTCTTCTTGGTGGTGGACGG	147
**A-H-specific loss**			
4q25	TATGGAACCTTCGCCTTCCTC	ATCAGCTTGTCCTGGGAATCA	150
5q21.3	GAACATGTATCGATTCGAGGGC	AAGCACCTACCTTAGGAATAGGATT	134
5q22.2	TCCCTGGAGTAAAACTGCGG	ACCCATAGGAACAGGACTGC	104
5q31.2	GAGGAATGCTGGCAATGAACA	TCCCCAAATCATGACTGTACCTG	117
5q33.3	GGAATTGGGGCAGAAATGAGC	TCACCATGGGAAAACGTCACT	117
9q34.12	GGAGATCTGCCTGAAGCTGG	CTACTCAGCCCAACCCGTG	107
9q34.3	AGTGCCTCTGACCACACTTC	TTCCCCAAGGGACCCTAGAA	145
9q22.33	TGGCTGTTAGGCTATTGGCTT	TGGCACACTACACACAGCAT	131
**A-L-specific gain**			
2q32.3	TGCCTTCCCACAGAGTTAAAAGA	TTATGTTTCACCCACCTGAGCC	147
2q32.2	ACACCATTGGTCTCGTGTTCT	ACCTGTCACTAGGCAGCATT	129
15q25.1	TGTTCTGGCAGTGTCTACGG	AGGTCCAAGCAAAACGTCCA	124
**S-H-specific gain**			
11q12.1	GCGAATACCATGTGACTC	ATCGGGCTTCTATCTACC	165
12p13.1	CAGCTTGCCCGAGTTCTACT	TGTCCTCAGAGTTAGCCGGA	137
12p12.1	CACAAAGAAAGCCCTCCCCA	CTGTGTTTCTCCCTTCTCAGGAT	150
19q13.32	GTCTCCAGAGTATCCCTGTCA	AGAGCAAGACTCCGTTCC	179
**S-H-specific loss**			
8p23.3	ACCTTTCTGCTGCGTGAC	GGACCTGAGTTTGATTTG	158
17p13.1	ATCTTATAGTTGCCTGTGGG	CTTCAATCTTAATTTGGGTG	199
**S-L-Specific gain**			
2p14	GTATCCCTTCTCCTCGTTTG	TCAGGACTGCTGTCACCC	155
3p21.31	TACAGGAAACCCATAGAA	ATAACCGTCTGAAACTCA	116
10q24.32	AGGTGTAAGGGCAAGAGTG	GAGTCAGCGAAGGCGATA	117
10q26.3	CCCTGATGCTTCCTGTTC	CACAAGTTTCCTCGATTACTG	118
15q11.2	AGTGAGCCTAGATTACACC	TATACTTGGCACCTTTGT	182
15q15.2	AGGGAAGCCACGACTCTG	TTCGGTCTTCACAACAATCC	216
15q22.31	GAGTGGCGGCTGATTGTT	TGGTGCTGGACTTCGTTC	168
**S-L-Specific loss**			
8p12	GCGGTGCGGTAAGTTCCT	CAGTGGCAAAGCCTAAGACAA	212
8p11.23	CTCGGCTGCCATCCTCCT	CCACCACCACCTTCCACA	242
**Candidate genes**			
DDB1	ACAGGGACAGCAGGTGGT	CTGCCTCTTCAGGATACACC	246
ERCC1	AAGGCACTGTCCATTCATC	GGAGGGTAGCAGCAGATT	102
ERCC2	GGTTTGAAGAGTGGTTGG	CTCATAGAATCGGCAGTG	177
PRPF19	CCAAGGGCTGACTCCACT	GAGACGGGCAATGACACG	185
BCAT1	CACTTCCAGCTTTCCCTT	GTACCAGAGCCAAACATC	234
CDKN1B	GAATAAGGAAGCGACCTG	AATCAGAATACGCCGAAA	122
MARK4	CCAGAAGGTCAAGCCACT	AACGCTCAGAAGAACAAA	197

Supplementary Table 2: Primers used for mRNA expression of the selected genes

Gene	Forward	Reverse	Amplicon/bp
MS4A2	GGCCTATATCCACATCCACAGT	TACCAAGTCCCAGAATGGTGA	116
CD5	TGACCTGCTTAGAACCCCAGA	GCTGCCGCTGTAGAACTCC	152
ERCC1	CTACGCCGAATATGCCATCTC	GTACGGGATTGCCCCTCTG	161
DDB1	ACCGGACACTTTACTTCGGC	TCGGCGGTGACCACATAGA	83
ERCC2	AGAAGGTGATTGAAGAGCTTCG	ACCTCAGGGTGAATACACAAGT	121
BCAT1	AGCCCTGCTCTTTGTACTCTT	CCAGGCTCTTACATACTTGGGA	103
PRPF19	CGAGAAGTACATTGCGGAGAAT	GCTGGTACAGAGCGTGTGAC	239
IFLTD1	TCCGAAAGCGTGTGTTTCAGT	CTTGAGCTTGTTCCACCTGAT	108
CDKN1B	TAATTGGGGCTCCGGCTAACT	TGCAGGTCGCTTCCTTATTCC	116
MARK4	TGAAGGGCCTAAACCACCC	CCAGCACTTGCGTACTCCA	94
TRAPPC6A	CTGGAGGGTATGGGGTTCC	TTGTCTTGCAGGACGTAGGTC	185
EXOC3L2	CGTGTGGAGCGATTCCATGA	GGCGATGGTCTTGCTGATATAG	75
CKM	ATGCCATTCGGTAACACCCAC	GCTTCTTGTAGAGTTCAAGGGTC	124
KLC3	GCCACGCTCAACAACTTGG	CGCTCCACGTCCTCAAACTT	188
PPP1R13L	TGCAACGACACAGTCATCTG	GCCCCATACTCTGCTCGAC	163
CD3EAP	AGATACGGAGCTGTGGCTTAT	CCCATTGAAGCATTCTGGGG	61
FOSB	GCTGCAAGATCCCCTACGAAG	ACGAAGAAGTGTACGAAGGGTT	249
RTN2	TCAGGGTTTACCGCAAAGTG	CTTGAGGGAATCCACGAGGTC	200
PPM1N	CGAGCGTTGGGCGACTTTA	CAGGAGCATGAACTCGTCCTC	123
VASP	ATGGCAACAAGCGATGGCT	CGATGGCACAGTTGATGACCA	147
OPA3	CCTATGGCGAAGCTGCTATAC	GGCGGCCTCCTTAATACGG	75
BCL2L14	CAAAATCCTCGCCTACTACACC	GACTCATTTGCTGAACAATTCCC	126
LRP6	TGTCAGCGAAGAAGCCATTAAA	TCTAAGGCAATAGCTCTGGGT	231
MANSC1	CACTAAGGCTGTCTGCTAGTCA	TCGAGTGTCGAAGATCATCAAGT	203
DUSP16	CCTGACTTTATCCCCGAGTCT	GAGATCCCAGCTAAACAGTGC	155
CREBL2	AGTAAAGAAGCCCGGTAAACG	CGACTGGATACCAACTCTTCCAA	144
GPR19	ATCTTCGGCAATTCCCTGGTT	GCAACGCTGATGAGAAGGTC	113
ST8SIA1	GTCCTCTGTTGGCTCTACATCT	CCCCGTCATACCACATGCTC	214
PPP1R37	CTCGACTGTCTGGACCTGAAA	GCCGAGGCACCATCTTCATC	134
MS4A8	GGCTTGAACATCGTCAGT	GCGTAAGGATAATAGTCG	107
MS4A15	TGTGGACAGGGGCTATCTGG	GAAGTGCATGGCAATGACCG	79
GAPDH	TGTTGCCATCAATGACCCCTT	CTCCACGACGTACTCAGCG	102

Supplementary Table 3: Details about primary antibodies used for Western blotting

Genes	Antibodies	Catalog No.	Supplier
MS4A2	Rabbit polyclonal to MS4A2	ab203747	ABCAM, Inc. UK
CD5	Anti-CD5 (26-40) antibody produced in rabbit	SAB1104201	Sigma-Aldrich, USA
ERCC1	Anti-ERCC1 antibody produced in rabbit	SAB4500795	Sigma-Aldrich, USA
DDB1	Anti-DDB1 antibody produced in mouse	SAB1405702	Sigma-Aldrich, USA
ERCC2	Monoclonal Anti-ERCC2 antibody produced in mouse	WH0002068M1	Sigma-Aldrich, USA
BCAT1	rabbit BCAT1 Antibody	12822	CST, USA
PRPF19	Anti-PRPF19 antibody produced in rabbit	SAB4501215	Sigma-Aldrich, USA
IFLTD1	ANTI-IFLTD1 (C-TERM) antibody produced in rabbit	SAB1303298	Sigma-Aldrich, USA
CDKN1B	p27 Kip1 (D69C12) XP® Rabbit mAb	3686	CST, USA
MARK4	rabbit MARK4 Antibody	4834	CST, USA
TRAPPC6A	Anti-TRAPPC6A antibody produced in rabbit	HPA043043	Sigma-Aldrich, USA
EXOC3L2	ANTI-EXOC3L2 (N-TERM) antibody produced in rabbit	SAB1302947	Sigma-Aldrich, USA
CKM	Anti-CKM (166-180) antibody produced in rabbit	C1869	Sigma-Aldrich, USA
KLC3	Anti-KLC3 antibody	ab168242	ABCAM, Inc. UK
PPP1R13L	Anti-iASPP antibody	ab34898	ABCAM, Inc. UK
CD3EAP	Anti-PAF49 antibody	ab76933	ABCAM, Inc. UK
FOSB	FosB (5G4) Rabbit mAb	2251	CST, USA
RTN2	Anti-Reticulon 2 antibody	ab103170	ABCAM, Inc. UK
PPM1N	Anti-PPM1N antibody	ab168300	ABCAM, Inc. UK
VASP	VASP (9A2) Rabbit mAb	3132	CST, USA
OPA3	Anti-OPA3 antibody	ab69163	ABCAM, Inc. UK
BCL2L14	Anti-Bcl G antibody	ab115467	ABCAM, Inc. UK
LRP6	LRP6 (C5C7) Rabbit mAb	2560	CST, USA
MANSC1	Anti-MANSC1 antibody produced in rabbit	HPA007955	Sigma-Aldrich, USA
DUSP16	Anti-DUSP16, C-Terminal antibody produced in rabbit	SAB4503215	Sigma-Aldrich, USA
CREBL2	Anti-CREBL2 (N-term K27) antibody produced in rabbit	SAB1300866	Sigma-Aldrich, USA
GPR19	Anti-GPR19 antibody produced in rabbit	SAB2900709	Sigma-Aldrich, USA
ST8SIA1	Anti-ST8SIA1 antibody	ab37806	ABCAM, Inc. UK
PPP1R37	Anti-PPP1R37 antibody produced in rabbit	HPA041500	Sigma-Aldrich, USA
MS4A8	Anti-MS4A8 antibody produced in rabbit	HPA007318	Sigma-Aldrich, USA
MS4A15	Mouse polyclonal to MS4A15	ab72697	ABCAM, Inc. UK

Supplementary Table 4: Shared and specific CNV regions for A-H and A-L.
See Supplementary_Table_4

Supplementary Table 5: Functional enrichment of genes in A-H- or A-L-specific CNV regions.
See Supplementary_Table_5

Supplementary Table 6: Shared and specific CNV regions for S-H and S-L .
See Supplementary_Table_6

Supplementary Table 7: Functional enrichment of genes in S-H- or S-L-specific CNV regions.
See Supplementary_Table_7

Supplementary Table 8: Analysis of genes identified in CNV regions shared by both highly and minimally invasive/migratory subclones and further amplified in one subclone.
See Supplementary_Table_8

Supplementary Table 9: Functional enrichment of genes identified in CNV regions in both highly or both minimally invasive/migratory subclones. See Supplementary_Table_9

Supplementary Table 10: Analysis of genes amplified in both highly invasive/migratory subclones in the TCGA ovarian cancer database (TCGA, Provisional) with the cBioPortal tool.
See Supplementary_Table_10
